# Eight Weeks of 448 kHz Radiofrequency Therapy Increases Multifidus Muscle Cross-Sectional Area and Modulates Thoracolumbar Range of Motion in Horses with Chronic Back Pain

**DOI:** 10.3390/ani16152437

**Published:** 2026-08-06

**Authors:** Marc Beaussart, Roland Perrin, Eva Marunova, Fanny Moraine, Delphine Fourteau, Roberta Blake

**Affiliations:** 1 School of Agriculture, Animal and Environmental Sciences, Anglia Ruskin University, Lordship Road, Writtle, Chelmsford CM1 3RR, UK; mjb162@pgr.aru.ac.uk (M.B.); eva.marunova@aru.ac.uk (E.M.); 2 Clinique Equine Desbrosse, Chemin de Mallebranche, Les Petites Yvelines, 78610 Les Bréviaires, France

**Keywords:** equine back pain, CSA, multifidus muscle, radiofrequency, range of motion

## Abstract

We aimed to investigate the effects of an 8-week 448 kHz radiofrequency (RF) on multifidus cross-sectional area (CSA) and thoracolumbar differential range of motion (ROM). Eight Thoroughbred horseball horses received four RF treatments (T9–L6) at 2-week intervals. Multifidus CSA (T10–L5) and differential spinal ROM between the withers (W), T13, T18, L3 and sacrum (S) were measured before treatment, immediately after the first session, 24 h after the first session, and at 8 weeks (15 days after the last session). Linear mixed models with Tukey post hoc comparisons were used. Significant effects of timepoint for CSA were significant with CSA increased at 8 weeks at all sites (T10–L5; all *p* < 0.001). No significant Time × Side interactions were identified for CSA (all *p* ≥ 0.334), demonstrating bilaterally consistent responses. Absolute increases versus baseline were greatest at T18 (+2.52 cm^2^), L5 (+1.90 cm^2^), and T16 (+1.77 cm^2^) (all *p* < 0.001). Axial rotation ROM increased transiently between W–T13 at 24 h (+4.8°, *p* < 0.001) but returned toward baseline at 8 weeks. Caudal thoracolumbar motion (T18–L3) decreased at 8 weeks (−4.2°, *p* = 0.004), suggesting improved segmental stability. Flexion–extension increased immediately post-treatment at W–T13 (+2.29°, *p* = 0.009) before reducing below baseline at 8 weeks (−2.49°, *p* = 0.005). Lumbosacral ROM showed minimal change. RF therapy was associated with multifidus hypertrophy alongside region-specific modulation of spinal motion, characterised by transient mobility increases followed by reduced caudal thoracolumbar motion, consistent with improved neuro-muscular dynamic stabilisation via multifidus hypertrophy.

## Simple Summary

Chronic back pain is a common problem in sport horses, and can reduce performance and welfare by weakening the muscles that help to support and stabilise the spine. This study investigated whether a form of deep heat therapy using radio waves could improve these muscles and influence how the back moves. Eight Thoroughbred horseball horses with long-term back pain received four treatment sessions over eight weeks. The size of a stabilising spinal support muscle was measured using ultrasound, and back movement was assessed using wearable motion sensors. The treated horses showed a clear increase in the size of the spinal muscle after the treatment period, together with changes in back movement that suggest improved spinal stability. These findings indicate that this therapy may help strengthen the muscles that support the spine and could become a useful addition to rehabilitation programmes for horses with chronic back pain. Improving spinal stability may enhance comfort, welfare, and potentially athletic performance, although larger studies are needed to confirm these findings and determine whether they lead to long-term reductions in pain and improved function.

## 1. Introduction

Back pain is a common cause of decreased performance in sport horses, regardless of their activity level [1]. Equine sports medicine is increasingly interested in the multifidus muscle, despite limited knowledge of its normal function in healthy horses [2]. The multifidus muscle has not been the subject of in-depth studies, and the effect of different training and rehabilitation exercises on its activity remains largely unknown [2]. However, the multifidus muscle has generated considerable interest in the equine literature due to the suggested links between its atrophy and axial pathologies of the spine [3,4].

The multifidus muscle is the main postural muscle responsible for intersegmental stabilization of the spine and contributes two-thirds of the total increase in spinal stability induced by muscle action [5,6,7]. Its fibres composition may vary slightly among breeds, but it appears to contain an equal proportion of class I and class IIA fibres [8,9]. In the horse, the multifidus muscle comprises five distinct multipennate fascicles traversing one to five intervertebral discs, which emanate from each spinous process and insert onto the mammillary process of the articular pillar of each thoracolumbar vertebra [6,7,10]. This established role of the muscle in global stabilization must be distinguished from the function of individual fascicles at the segmental level, which has not yet been determined [2,9]. The multifidus muscle prevents abnormal rotation during contraction of antagonist muscles, being activated in anticipation with the transversus abdominis muscle to stabilize the spine before and during body movement [8,11]. Bone or joint pathologies induce selective ipsilateral atrophy of the multifidus muscles at the same vertebral level. This causes spinal instability. The epaxial musculature then contracts through compensatory mechanisms, leading to stiffness or hypertonia [3,11,12,13].

Ultrasound evaluation typically includes examination of the joints of the articular processes as well as the multifidus muscle [3,14,15]. The ultrasound technique used in this study has been validated by studies evaluating the right-left symmetry of the cross-sectional area (CSA) of the multifidus muscle in the thoracolumbar region [16,17]. Thus, systematic measurement of the multifidus muscle CSA provides an objective means of post-treatment assessment [18]. Similar results, showing a clear correlation between spinal pathology and progressive atrophy of the multifidus and longissimus muscles have been described in previous studies [3,17,18,19]. Atrophy and asymmetry of the multifidus and longissimus dorsi muscles have been associated with back pain in horses [3,11]. However, it has not yet been established in horses whether this laterality of epaxial muscle atrophy directly contributes to the alteration in locomotion or if the atrophy/asymmetry is cause or effect of back pain. Horses with back pain exhibit abnormal back movements, which, through objective measurement techniques, allow for the detection of dorsal dysfunction [20]. Conversely, effective treatment of back pain results in a return to normal, characterized by an increase in ROMs and improved symmetry at the trot [21]. A significant difference in back range of motion (ROM) has been demonstrated between asymptomatic horses and horses with back pain [20]. Flexion (FE), lateral bedding (LB), and axial rotation (AR) are limited in horses with back pain.

Heat therapy is a widely used tool in physiotherapy for the management of various physiological and pathological conditions in equine athletes [22]. Heat therapies decrease tissue viscosity and increase tissue elasticity [23]. Radiofrequency (RF) technology works by diathermy, a therapeutic process that uses electrically generated heat to penetrate the body. The capacitive (CAP) system targets soft tissues and muscles, while the resistive (RES) system targets larger, more resistant tissues such as tendons, bones, and joints. RF devices use electromagnetic waves with frequencies ranging from 3 kHz to 300 GHz. Depending on the structure being treated, a CAP or RES electrode is used (active electrode), in conjunction with a second electrode attached to the patient (inactive electrode). The effects of RF are associated with two types of actions: electrical and thermal [24]. The CAP and RES increase the temperature of the targeted organs. Blood circulation dissipates heat to adjacent areas, thus maintaining the temperature of the treated structure within the desired range and preventing undesirable tissue hyperthermia [13,25]. RF also promotes endothermy, leading to increased superficial and deep blood circulation, oxyhemoglobin levels, and improved ROM, flexibility, and functional mobility [24,25]. Finally, RF stimulates the cell signalling pathway that promotes the proliferation of mesenchymal stem cells. This results in tissue regeneration by activating stem cells present in the injured area [25,26,27]. However, most of the therapeutic effects of RF in horses have not yet been studied or objectively evaluated [13].

This study explored the effects of RF on back pain in horses, based on two aims. The first aim was to determine whether RF treatment increases the CSA of the multifidus muscle in the short or/and medium term, and consequently. The second was to observe the effect of RF on back ROM in horses suffering from chronic back pain in the short and medium term.

## 2. Materials and Methods

**Figure 1:**
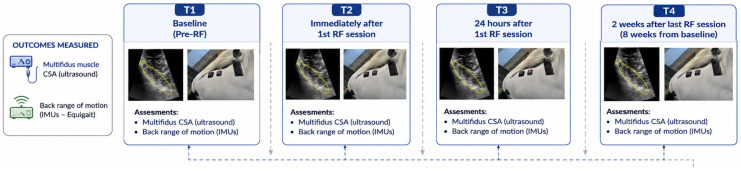
Study design and assessment timeline. Horses underwent a prospective longitudinal repeated-measures study evaluating the effects of radiofrequency (RF) treatment on multifidus muscle cross-section area and back ROM. Assessments were performed at four time points: T1, baseline (pre-RF treatment); T2, immediately following the first RF session; T3, 24 h after the first RF session; and T4, 15 days after completion of the fourth and final RF session (8 weeks). The RF treatment protocol consisted of four sessions delivered at two-week intervals (week 0, week 2, week 4 and week 6). At each assessment, multifidus muscle cross-sectional area (CSA) was measured using ultrasonography, and back ROM were evaluated using inertial measurement units. Each horse served as its own control throughout the study.

A prospective longitudinal repeated-measures study design was employed to evaluate changes in multifidus muscle CSA following radiofrequency treatment. Measurements were obtained at baseline (pre-treatment) (T1), immediately after the first radiofrequency session (T2), 24 h after the first session (T3), and 2 weeks following completion of four radiofrequency sessions protocol (T4). In the absence of a control group, each horse acted as its own control, allowing assessment of temporal changes in multifidus CSA in response to treatment. This approach is consistent with previously published equine multifidus studies that have evaluated changes in muscle CSA and back ROM over time using repeated measurements within the same individuals [1,2] (Figure 1).

### 2.1. Ethical Approval

Ethical approval was provided by the School of Agriculture, Animal, and Environmental Sciences Research Ethics Panel, reference number ETH 2425-9058; approval date, 19 August 2025. Informed owner consent was obtained.

### 2.2. Horses

The horses included in this study were 8 adult English Thoroughbreds (*n* = 8), with clinical back pain and overriding dorsal spinous processes diagnosis, used by a national horseball team competing in the professional pro elite male and female categories. The horses were a median age of 12 years (range: 9–15 years), a median bodyweight of 485 kg (range: 475–520 kg), and a median height at the withers 160 cm (range: 158–166 cm). (*n* = 3) were geldings and (*n* = 5) were mares. Inclusion criteria included: (a) Horses considered to have been suffering from back pain of varying intensity for at least 4 months, based on the history provided by the owners and veterinary history, previous imaging (radiographs) confirming overriding dorsal spinous processes. (b) Horses who underwent veterinary diagnosis by two qualified veterinarians (DF and FM) during the enrolment to this study, confirming the presence of pain in the longissimus dorsi muscle upon palpation of the thoracolumbar region. (c) Horses judged to be sound and with no clinical lameness or ataxia observed during examination by two qualified veterinarians. (d) Horses who maintain the same training intensity with the same riders. (e) Horses free from any systemic or local administration of anti-inflammatory drugs during the 8 weeks before the study. The horses were assigned numbers to conceal their identity.

### 2.3. Radiofrequency

A RF device (TECAR VET 4000, Globus, Codogne, Italy) was used and applied by a qualified veterinary physiotherapist (MB). The device consists of a rigid circular metal active electrode (70 mm in diameter) and a flexible rectangular metal plate serving as the inactive electrode. The inactive electrode was placed on the sternum. A conductive gel (Globus, Codogne, Italy) was applied as a coupling medium between the active electrode and the skin surface as well as between the inactive electrode and the skin surface. The RF protocol consisted of applying CAP and RES to the left and right sides (balanced 50/50) [13,22], starting with one side randomly. The session combined the use of both electrode types, CAP and RES, between T9 and L6. The active electrodes were 70 mm in diameter, chosen due to the extended treated area [22]. The settings were as follows: frequency at 448 kHz, 5 min of 50% CAP, followed by 12 min of 50% RES, then 3 min of 12% CAP on each side, for a total of 40 min [13]. It should be noted that there is no basis for optimal dosage or choice of mode [28]. Four radiofrequency sessions were performed, spaced 2 weeks apart.

### 2.4. Ultrasonography

Ultrasonography was performed with the horses standing square and restrained in stocks. Ultrasonographic examinations were performed by two qualified veterinarians (DF and FM) experienced in equine musculoskeletal ultrasonography. Both veterinarians performed the ultrasonographic examinations throughout the study, to ensure consistency. The scans were acquired solely for standardized anatomical measurements of multifidus muscle cross-sectional area and were not used for diagnostic evaluation of spinal pathology. The ultrasound device was the IQ + VET (Butterfly, Burlington, MA, USA) connected by a USB cable to an iPad Air (Apple, Cupertino, CA, USA) used to view the images and to store them. The thoracolumbar region was already clipped because the horses are in training. The dorsal spinous processes were identified by combining palpation and ultrasonography. A razor mark was performed on the horses after the first measurement to ensure the reliability of anatomical landmarks, to avoid errors in placement of the ultrasound probe, and to ensure conclusive repeatability. After standard skin preparation, alcohol was applied to allow ultrasound observation. For the measurement immediately after radiofrequency application, a microfiber cloth was applied to remove any remaining contact cream used for radiofrequency. Ultrasound images were acquired bilaterally at the levels of T10, T12, T14, T16, T18, L2 and L5. These choices of anatomical landmarks (vertebral bone anatomy [29]) are taken from other studies using CSA ultrasound to observe the multifidus [9,14,17,29,30,31,32,33,34]. The authors increased the number of measurement points compared to other studies carried out, having neither consensus nor universal list in the scientific literature available at the time of carrying out this study. The probe was oriented transversely adjacent to the dorsal midline and followed the skin curvature of the epaxial muscles with an angle of approximately 45° [17]. Images were captured with the probe placed perpendicular to the skin and the dorsal spinous processes of the vertebra [29], following the anatomical landmarks: dorsally (the spinous process) and ventrally (the bony margin of the rib or transverse process). At each measurement, three images per anatomical landmark were captured, on both the left and right sides, to account for slight variations in position or angulation of the probe [17]. Measurements were acquired on still, recorded image via Image J software (v1.54p) (ImageJ.NIH.gov) in cm^2^. Four measurements were performed, at T1, T2, T3 and T4.

### 2.5. Differential Range of Motion (ROM)

Each horse was equipped with a 9-sensor inertial measurement unit (IMU) gait analysis system (EquiGait Ltd., Waltham Cross, UK) using a MTw-Awinda setup (Xsens, Enchede, The Netherlands). Each IMU contained a tri-axial accelerometer (±16× gravity), a tri-axial gyroscope (±2000 deg/s) and a tri-axial magnetometer (±1.9 Gauss), with dynamic accuracy 0.75 degrees root mean square for orientation output (manufacturer specifications). The 9 IMU sensors were attached with double-sided tape and custom-made Velcro pouches to the horse [35,36]. Inertial sensor displacement and symmetry data show acceptable accuracy and good levels of consistency for back movement [35]. Anatomical landmarks were used to place the sensor over the poll, over the withers (W), over T13 and T18, over L3, over both left and right tuber coxae, over the sacrum (S) (caudal) and over the tail (cranial). Data collection was initiated manually, with an operator starting recording from the EquiGait software (v1, EquiGait Ltd., Waltham Cross, UK) communicating with the IMUs via Bluetooth Low Energy (BLE) connection, logging data onto the onboard memory at a sample rate of 60 Hz per individual channel. The analysis and extraction of the differential ROM was performed by the same software, EquiGait. (v1, EquiGait Ltd., Waltham Cross, UK). Data were collected at in-hand trot for a total of 240 m (4 × 60 m) on a dirt surface. Likewise, four measurements were performed, at T1, T2, T3 and T4.

### 2.6. Data and Statistical Analysis

All analyses were performed using jamovi (v2.6.44), using the GAMLj module. Linear mixed-effects models were used. Separate models were constructed for each vertebral level for multifidus cross-sectional area (CSA; T10, T12, T14, T16, T18, L2, L5) and for each thoracolumbar segment for differential range of motion (ROM) in axial rotation (AR), flexion–extension (FE), and lateral bending (LB) between W–T13, T13–T18, T18–L3 and L3–S. Time was included as a fixed effect in all models. For CSA, Side (left/right) and Time * Side interaction were also included. Horse was included as a random intercept. Model fit was assessed using likelihood ratio tests. Marginal and conditional R^2^ values were calculated. Post hoc pairwise comparisons were adjusted using Tukey correction. Significance was set at *p* < 0.05.

## 3. Results

None of the horses showed any adverse effects during the study and all horses successfully completed the study. Descriptive statistics can be found in the Supplementary Tables S1 and S2.

### 3.1. Multifidus Cross-Sectional Area

**Figure 2:**
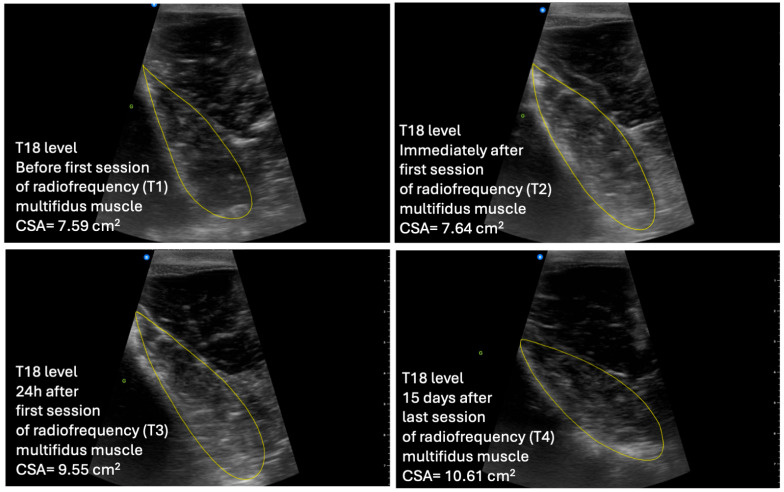
Representative ultrasonographic images of the multifidus muscle at the T18 vertebral level from the same horse across the four assessment time points: T1, baseline (before the first radiofrequency [RF] treatment session); T2, immediately after the first RF treatment session; T3, 24 h after the first RF treatment session; and T4, 2 weeks after the fourth and final RF treatment session (8 weeks). The yellow outline delineates the multifidus muscle cross-sectional area (CSA), with the measured CSA displayed for each time point. This representative example illustrates the progressive increase in multifidus muscle CSA observed following the RF treatment protocol.

Figure 2 shows an example of multifidus CSA measurements at T18 from the same horse across all timepoints.

**Figure 3:**
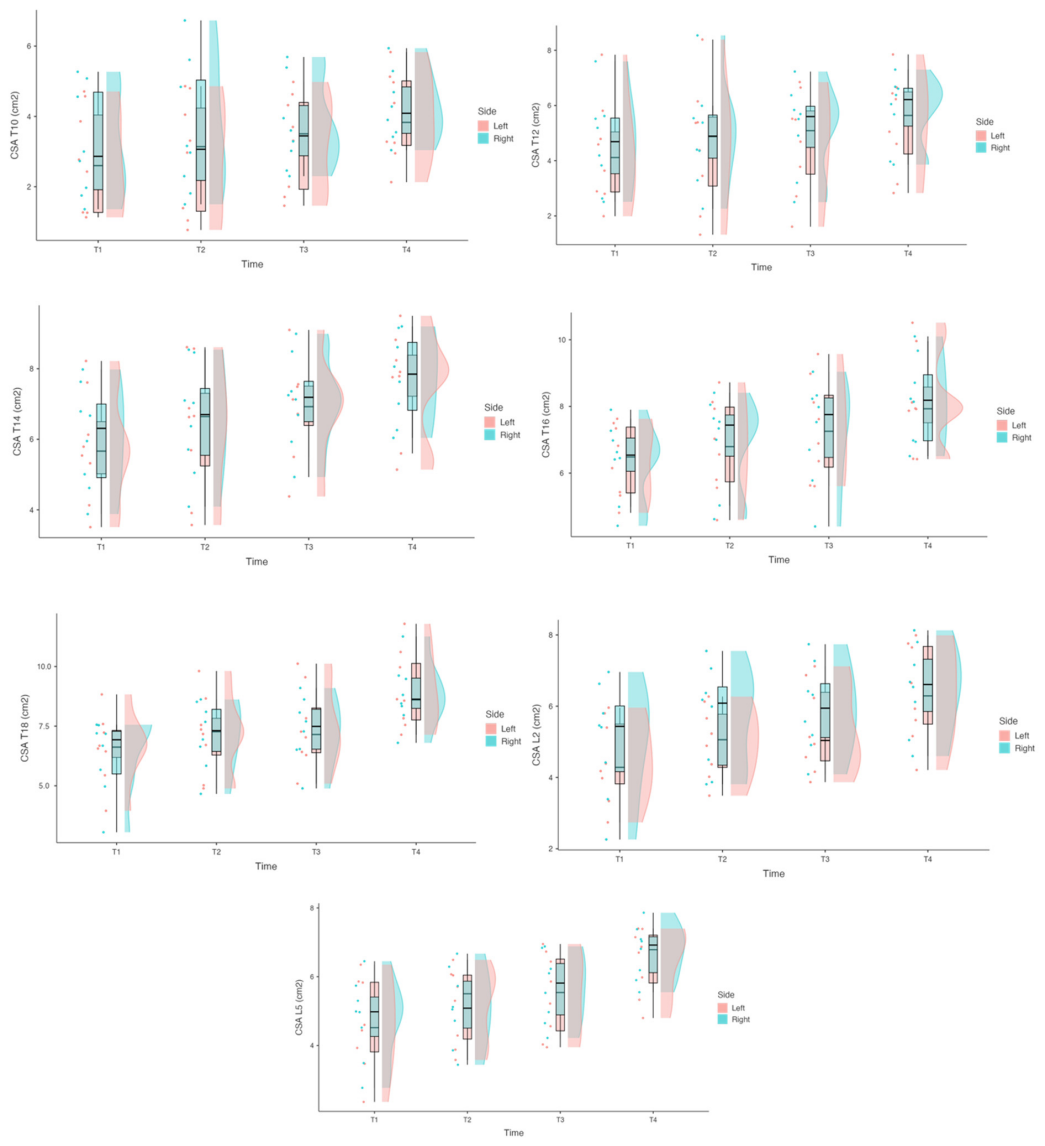
Distribution of multifidus muscle cross-sectional area (CSA) measured by ultrasonography at vertebral levels T10, T12, T14, T16, T18, L2 and L5 across the four assessment time points (T1, baseline; T2, immediately after the first radiofrequency (RF) treatment session; T3, 24 h after the first RF treatment session; and T4, 15 days after the fourth and final RF treatment session). Data are presented separately for the left (pink) and right (blue) sides. Individual data points represent measurements from individual horses, boxplots display the median and interquartile range (IQR), whiskers indicate the data spread, and half-violin plots illustrate the distribution of CSA values at each time point.

Linear mixed-effects models demonstrated significant effects of time on multifidus cross-sectional area (CSA) at all thoracic and lumbar sites (all *p* < 0.001) (Figure 3). Model fit was strong across levels, with conditional R^2^ values ranging from 0.805 to 0.933, indicating that combined fixed and random effects explained a substantial proportion of variance. Intraclass correlation coefficients ranged from 0.75 to 0.92, demonstrating considerable between-horse variability in baseline CSA measurements. CSA increased progressively over time, with the largest values consistently observed at T4. Compared with T1, CSA at T4 was significantly greater at T18 (+2.52 cm^2^, *p* < 0.001), L5 (+1.90 cm^2^, *p* < 0.001), T16 (+1.77 cm^2^, *p* < 0.001), T14 (+1.75 cm^2^, *p* < 0.001), L2 (+1.68 cm^2^, *p* < 0.001), T12 (+1.12 cm^2^, *p* < 0.001), and T10 (+1.14 cm^2^, *p* < 0.001). T2 and T3 measurements showed limited or inconsistent differences relative to baseline, indicating that CSA increases developed progressively throughout the intervention period rather than occurring acutely following treatment (Figure 2). No significant Time × Side interactions were detected at any vertebral level, demonstrating bilaterally consistent adaptations. A modest main effect of side was observed at T10, T12, T14 and L2, where right-sided CSA values were slightly greater than left (*p* ≤ 0.032); however, this trend remained stable across time and did not influence treatment responses.

### 3.2. Differential Range of Motion

Time effects differed according to spinal region and movement plane. In axial rotation, a significant temporal effect was observed between the withers and T13 (W–T13; *p* < 0.001), with ROM increasing T3 compared with T1 (+4.8°, *p* = 0.002) before returning toward baseline values at T4. A significant time effect was also detected at T13–T18 (*p* = 0.012), where post hoc comparisons indicated reduced motion at T4 relative to T3 (*p* = 0.011). Caudal thoracolumbar axial rotation (T18–L3) decreased significantly at T4 compared with T1 (−4.2°, *p* = 0.004). In contrast, axial rotation at the lumbosacral region (L3–S) did not demonstrate a significant overall time effect (*p* = 0.087), although small increases were observed at intermediate time points. Flexion–extension ROM demonstrated significant temporal changes across cranial and mid-thoracolumbar segments. Between W–T13, flexion–extension increased immediately following treatment (T2 vs. T1: +2.29°, *p* = 0.009) but was reduced below baseline values at T4 (−2.49°, *p* = 0.005). Similar patterns were observed at T13–T18 and T18–L3, where motion increased immediately post-treatment before decreasing at later time points. No significant temporal effect was identified at L3–S (*p* = 0.231). Lateral bending showed no significant time effects across thoracolumbar segments (all *p* > 0.05), with only minor transient fluctuations that did not persist over the study period.

## 4. Discussion

To our knowledge, this is the first study evaluating the effects of RF on the multifidus muscle in horses suffering from back pain, while simultaneously measuring back ROM. This study demonstrated that an 8-week 448 kHz RF protocol was associated with increased multifidus muscle CSA in horseball horses with chronic back pain. Concurrently, thoracolumbar spinal motion exhibited region-specific modulation characterised by transient increases in mobility followed by reduced caudal thoracolumbar motion at 8 weeks.

In the human scientific literature, multifidus muscle atrophy has been observed in patients with back pain, in both acute and chronic cases. In cases of unilateral pain, the atrophied side is ipsilateral and localized to the painful area [30,31,32]. Inactivity of the multifidus muscle leads not only to muscle atrophy, but also to fat infiltration. This decreases its activation and induces a vicious cycle of increased pain and muscle dysfunction [34]. Furthermore, in cases of multifidus muscle injury, it does not regain its full strength or endurance without specific exercises that can reactivate the appropriate motor response [29,37]. Data from the human literature suggest that rest or pain relief alone does not automatically lead to reactivation or hypertrophy of the multifidus muscle [37]. However, the equine and human back differ, particularly in spinal orientation, weight-bearing distribution and locomotion (quadrupedal versus bipedal), making direct translation of these findings between species challenging. In horses suffering from back pain, lameness, vertebral bone pathologies, and postural instability, a left–right asymmetry and atrophy of the multifidus muscle has been observed [3,7,29,34]. However, the exact nature of these relationships remains unknown [7]. Furthermore, in a group of Thoroughbred racehorses, a link was established between severe bone lesions of the spine and multifidus muscle atrophy on the more affected side. This atrophy was precisely localized to the vertebral lesion [3]. Finally, in horses, multifidus muscle asymmetry is also observed in cases of unilateral back pain [3,38].

All the horses observed in this study showed clinical signs of back pain and all had a previous imaging and clinical diagnosis of overriding dorsal spinous processes. For most horses, historical medical records indicated varying grades and locations of this condition, although complete grading data were not available for every horse. The caudal thoracic and lumbar spinal regions are most implicated in the development of pathologic processes such as overriding dorsal spinous processes [2,3,39]. In the present study, the observed increase in multifidus muscle volume in these regions in the short and medium term may indicate an increase need to spinal stability and consequent changes in mobility, consistent with previous findings [2]. Horses undergoing rehabilitation after medical or surgical treatment for overriding dorsal spinous processes have increased CSA measurements of the multifidus muscle, with a greater increase after surgical treatment [40]. The CSA findings suggest progressive muscular adaptation rather than acute treatment-related swelling, as increases were most pronounced at T4 rather than T2 or T3. The largest increases were observed at T18 and L5, regions subject to high mechanical demand and commonly implicated in equine thoracolumbar dysfunction. Restoration of multifidus mass at these sites may enhance segmental stability and load transfer.

In humans, RF is a technology that provides significant improvements in pain and injury (bone, muscle, and joint) [41]. RF promotes tissue regeneration and also has an analgesic effect. Its main mechanism of action is believed to be temperature elevation [42]. It is established that the most significant effects of RF on pain and muscle performance are primarily related to its thermal effect [23]. This temperature elevation has been described at a superficial level (10–15 mm/subcutaneous adipose tissue and fascia) and at a deep level (20–30 mm/muscle layer) [23,25,42,43]. Due to a lack of precise knowledge regarding the effects of RF in horses and the possible exchanges or transfers between tissues and fluids induced by the thermal effect, a CAP and RES protocol was implemented to best estimate the effects on the multifidus muscle [13,42]. However, it is impossible to determine, from a histological point of view, the direct effects on the multifidus muscle and the indirect effects originating from adjacent tissues.

An increase in local tissue temperature is associated with a reduction in local pain [41], a relationship attributed to thermal activation of nerve fibres, which reduces pain transmission [44] and thereby allows for an increase in ROM. There is also a change in muscle flexibility, linked to the increase in temperature, the increase in collagen, and the reduction in the viscosity and viscoelasticity of the connective tissue of muscle fibres [45]. Regarding the effects of RF on thoracolumbar pain, a study using accelerometers and based on the hypothesis that horses suffering from back pain had a reduced dorsoventral FE ROM compared to healthy horses showed that these ROMs increased after RF treatment [13].

According to the scientific literature, the thermal effect of RF should reduce pain and, consequently, increase ROM in the back of horses suffering from back pain. A horse with back pain exhibits limited ROM in FE, LB, and AR [20]. This study suggests that the ROM in FE between the withers and L3 increased significantly in the short term after a single RF treatment as well as other absolute ROM in AR and LB. However, in the medium term, after four RF treatments, the ROM in FE between the withers and the sacrum and the ROM in LB between the withers and T13 decreased considerably. Only a few absolute ROM values remained above the baseline values. This suggests that the short-term effect of RF increases mainly FE ROM of the back, but not in the medium term. This phenomenon occurred in parallel with significant growth of the multifidus muscle. The multifidus muscle typically atrophies in horses with back pain [11]. An increase in its volume is therefore a positive sign in the rehabilitation process and in reducing back pain. Its primary role is postural, responsible for intersegmental stabilization of the spine [5,6,7]. It also limits accessory movements during flexion and associated rotation [10]. Consequently, it is plausible that an increase in multifidus muscle volume improves spinal stabilization, thereby reducing accessory movements and the horse’s back mobility. Increased stability has been shown in the literature to reduce certain ROMs in horses [46,47]. Because horses compensate for their loss of intervertebral instability by increasing the tension of the longissimus dorsi muscle [11], it is also conceivable that increased spinal stability through the multifidus muscle could decrease the need for longissimus dorsi muscle tension. ROM findings suggest a biphasic response. Early increases in axial rotation and flexion–extension (particularly W–T13 and T18–L3) may reflect reduced nociceptive inhibition or increased tissue compliance immediately following treatment. In contrast, reduced axial rotation at T18–L3 at 8 weeks may indicate improved active stabilisation secondary to multifidus hypertrophy. Similar patterns are described in human low back pain rehabilitation, where improved deep stabiliser recruitment reduces excessive intersegmental motion. Lumbosacral motion (L3–S) remained largely unchanged, suggesting that RF effects were most pronounced in thoracic and cranial lumbar segments.

The limitations of this study stem primarily from the relatively small number of horses included. Recruiting a larger cohort while maintaining a consistent training programme and minimising confounding factors related to management and exercise was challenging. Consequently, the study had limited statistical power, and caution should be exercised when generalising the findings to the wider equine population. Nevertheless, the consistent direction of changes in multifidus CSA and region-specific thoracolumbar ROM supports a treatment-associated effect. In addition, although no separate control group was included, the repeated-measures design allowed each horse to serve as its own control, reducing inter-individual variability.

Ultrasonography is a reliable, non-invasive, objective, and sequential method for measuring CSA of spinal stabilizing muscles, particularly the multifidus, in both humans and horses, especially when performed by the same technician [29,48,49]. However, one study [14] highlighted the limitations of measuring the CSA of the multifidus by ultrasound: measurements taken over several days, the use of different devices, and difficulties in reproducibility depending on the location. Although a portable ultrasound system was used, the latest-generation device provided image quality that was considered appropriate for the acquisition of standardized anatomical CSA measurements. The pressure exerted on the probe can also vary from one side to the other [49]. To minimise these sources of variability, all examinations were performed by the same two experienced operators throughout the whole study using the same ultrasound unit and a standardised scanning protocol.

The variability of clinical signs of back pain may also be a limitation. It is clear that once overriding dorsal spinous processes are diagnosed, back pain is considered chronic, and therefore its onset can be difficult to determine. The horses studied were Thoroughbreds, a breed predisposed to overriding dorsal spinous processes [50]. Overriding dorsal spinous processes syndrome remains the most frequent cause of back pain in horses [18,51]. However, the clinical signs associated with this condition are variable, and a weak correlation between radiographic findings and clinical manifestations has been described [13,52].

Another limitation, frequently noted in RF studies [13,42], is the lack of measurement of superficial and deep temperature variations in the treated tissues. Regarding surface temperature variations, changes were confirmed for the medium and high intensity protocols [22]. It would have been interesting to measure these variations at different tissue depths to understand the exact effect of RF on the multifidus muscle. However, this invasive protocol was impossible on sport horses at work. To date, no standardized treatment protocol has been reported for the clinical application of RF, either in horses or humans [53,54]. In this study, a protocol was established randomly regarding the RF sessions frequency and inspired by a study [13] concerning RF dosage. This study was conducted on horses participating in horseball, a specific and still relatively unstudied equestrian discipline. Back pain is common in horseball horses because this discipline mainly uses Thoroughbreds retired from racing due to loss of performance or injuries and which have significant genetic predisposition factors. In addition, the horse’s back is subjected to considerable stress during training and competitions, particularly when the rider has to retrieve the ball from the ground, make tight turns and sudden stops.

Studies on the effects of RF therapy in horses are scarce. In the future, it would be relevant to define different protocols regarding the dosage (number of applications and duration) of RF treatment, as well as its use at frequencies above and below 448 kHz. The vast majority of studies use 448 kHz because most RF devices do not allow for frequency adjustment. Histological observations of muscle fibers would also be valuable to better understand the short- and medium-term effects. Finally, it would be worthwhile to compare RF therapy with other complementary therapies that use heat as their mechanism of action.

Chronic thoracolumbar pain in sport horses is frequently associated with multifidus atrophy and impaired spinal stability. The present findings demonstrate consistent and significant increases in multifidus cross-sectional area following an 8-week 448 kHz RF protocol in Thoroughbred horseball horses. The bilateral hypertrophic response across thoracic and lumbar regions suggests that RF therapy may represent a useful adjunctive modality within multimodal rehabilitation programmes aimed at restoring paraspinal muscle mass and dynamic spinal support. Importantly, the greatest increases were observed at T18 and L5—regions commonly implicated in equine thoracolumbar dysfunction—supporting potential clinical utility in horses with caudal thoracic and lumbosacral pain syndromes. Further controlled trials are warranted to determine whether morphological improvements translate into measurable improvements in performance, kinematics, or pain-related behaviours.

## 5. Conclusions

This study demonstrated that 448 kHz RF therapy was associated with significant increases in multifidus muscle cross-sectional area and modulation of thoracolumbar range of motion in Thoroughbred horseball horses following an 8-week treatment protocol. These findings suggest that radiofrequency may represent a useful adjunctive therapy for enhancing spinal muscle morphology and promoting functional spinal stability. However, given the small sample size and absence of a control group, the findings should be interpreted with caution. Further controlled studies with larger populations are required to confirm these results and determine whether the observed morphological changes translate into meaningful improvements in pain, function, athletic performance, and long-term clinical outcomes.

## Data Availability

The original data presented in the study are openly available on Mendeley Data at the link https://data.mendeley.com/datasets/f94sw5rw9x/1 (accessed on 27 June 2026).

## Supplementary Materials

The following supporting information can be downloaded at https://www.mdpi.com/article/10.3390/ani16152437/s1. File S1: Descriptive statistics; File S2: Inferential statistics.
