# Supplementary material for: Eight Weeks of 448 kHz Radiofrequency Therapy Increases Multifidus Muscle Cross-Sectional Area and Modulates Thoracolumbar Range of Motion in Horses with Chronic Back Pain
Source: Animals (Basel). 2026 Aug 6;16(15):2437. doi: 10.3390/ani16152437 (PMC13465300; doi:10.3390/ani16152437)
Supplement: Supplementary file 1 [file animals-16-02437-s001.zip › Supplementary file S1.pdf]

Supplementary file S1: Descriptive statistics

Table S1. Descriptives for CSA

Descriptives

	Side	Time	Mean	SD
T18	Left	T1	6.59	1.452
		T2	7.15	1.665
		T3	7.45	1.685
		T4	9.01	1.629
	Right	T1	6.23	1.583
		T2	7.13	1.385
		T3	7.34	1.348
		T4	8.84	1.321
T16	Left	T1	6.38	1.099
		T2	6.79	1.427
		T3	7.35	1.485
		T4	8.15	1.463
	Right	T1	6.38	1.153
		T2	6.95	1.385
		T3	7.25	1.537
		T4	8.16	1.307
T14	Left	T1	5.78	1.584
		T2	6.32	1.875
		T3	6.82	1.415
		T4	7.59	1.498
	Right	T1	6.07	1.464
		T2	6.54	1.567
		T3	7.10	1.301
		T4	7.75	1.232
T12	Left	T1	4.29	1.895
		T2	4.55	2.298
		T3	4.66	1.854
		T4	5.35	1.762
	Right	T1	4.64	1.700
		T2	4.97	1.895

Descriptives

	Side	Time	Mean	SD
		**T3**	5.16	1.586
		**T4**	5.82	1.265
**T10**	**Left**	**T1**	2.75	1.490
		**T2**	2.90	1.657
		**T3**	3.26	1.383
		**T4**	3.99	1.276
	**Right**	**T1**	3.21	1.555
		**T2**	3.62	1.900
		**T3**	3.71	1.254
		**T4**	4.26	0.993
**L2**	**Left**	**T1**	4.47	1.162
		**T2**	5.01	1.002
		**T3**	5.36	1.233
		**T4**	6.37	1.372
	**Right**	**T1**	5.04	1.601
		**T2**	5.67	1.434
		**T3**	5.94	1.229
		**T4**	6.51	1.235
**L5**	**Left**	**T1**	4.61	1.359
		**T2**	5.17	1.107
		**T3**	5.47	1.204
		**T4**	6.45	0.979
	**Right**	**T1**	4.78	1.185
		**T2**	5.11	1.116
		**T3**	5.68	0.999
		**T4**	6.73	0.784

Table S2. Descriptives for ROM

Descriptives

	Time	Mean	Median	SD	IQR
**AR WT13**	**T1**	10.96	11.15	2.538	3.150
	**T2**	12.43	12.45	3.140	3.125
	**T3**	15.76	15.55	2.743	1.950
	**T4**	9.99	9.40	2.849	4.025
**AR T13T18**	**T1**	8.68	8.80	4.272	7.100
	**T2**	10.99	11.90	3.548	2.175
	**T3**	12.55	10.70	6.266	7.600
	**T4**	5.26	5.50	1.188	0.750
**AR T18L1**	**T1**	10.93	10.05	3.502	5.850
	**T2**	9.75	8.95	2.125	3.525
	**T3**	11.06	11.15	2.155	1.250
	**T4**	6.72	6.25	3.014	3.050
**AR L3S**	**T1**	9.36	9.85	2.417	2.600
	**T2**	9.94	10.75	3.460	5.100
	**T3**	12.13	12.20	1.951	2.625
	**T4**	12.15	12.50	3.492	4.250
**FE WT13**	**T1**	7.06	7.25	1.704	1.475
	**T2**	9.35	9.00	2.340	3.050
	**T3**	6.90	6.90	1.020	1.575
	**T4**	4.58	4.40	1.421	1.550
**FE T13T18**	**T1**	4.30	4.20	1.596	2.175
	**T2**	7.51	7.95	2.213	2.150
	**T3**	7.11	6.45	3.346	4.925
	**T4**	2.61	2.35	0.642	0.900
**FE T18L1**	**T1**	5.06	5.00	1.673	2.275
	**T2**	8.72	8.45	2.125	1.675
	**T3**	7.10	7.55	3.120	4.875
	**T4**	3.69	3.60	1.585	2.300
**FE L3S**	**T1**	7.74	8.40	2.086	1.725

Descriptives

	Time	Mean	Median	SD	IQR
	**T2**	7.30	7.10	2.669	3.225
	**T3**	8.63	8.35	2.540	2.850
	**T4**	6.17	6.35	1.940	2.000
**LB WT13**	**T1**	6.67	6.20	2.749	2.425
	**T2**	8.07	8.20	1.102	0.850
	**T3**	8.03	8.50	1.642	2.150
	**T4**	5.91	5.70	1.425	1.225
**LB T13T18**	**T1**	3.31	3.15	1.271	2.350
	**T2**	3.80	3.70	0.946	0.925
	**T3**	2.80	2.70	1.318	0.775
	**T4**	3.10	2.85	0.959	0.925
**LB T18L1**	**T1**	3.09	2.80	1.328	1.100
	**T2**	3.81	3.60	0.846	0.625
	**T3**	3.85	3.90	1.017	1.025
	**T4**	2.91	2.95	0.872	1.325
**LB L3S**	**T1**	2.38	2.30	0.727	0.875
	**T2**	2.80	2.95	0.965	1.500
	**T3**	3.24	3.05	0.921	1.050
	**T4**	2.50	2.65	0.496	0.525
