# Supplementary material for: Eight Weeks of 448 kHz Radiofrequency Therapy Increases Multifidus Muscle Cross-Sectional Area and Modulates Thoracolumbar Range of Motion in Horses with Chronic Back Pain
Source: Animals (Basel). 2026 Aug 6;16(15):2437. doi: 10.3390/ani16152437 (PMC13465300; doi:10.3390/ani16152437)
Supplement: Supplementary file 1 [file animals-16-02437-s001.zip › Supplementary file S2.pdf]

Supplementary file S2: Statistics outputs for the Linear mixed models

CSA T18

Model Fit

Type	R^2	df	LRT X^2	p
Conditional	0.874	8	105.505	<.001
Marginal	0.273	7	74.766	<.001

Fixed Effects Omnibus Tests

	F	df	df (res)	p
**Time**	45.100	3	49.0	<.001
**Side**	1.102	1	49.0	0.299
**Time ✻ Side**	0.206	3	49.0	0.892

Parameter Estimates (Fixed coefficients)

				95% Confidence Intervals				
**Names**	**Effect**	**Estimate**	**SE**	**Lower**	**Upper**	**df**	**t**	**p**
(Intercept)	(Intercept)	7.4665	0.493	6.477	8.456	7.00	15.133	<.001
Time1	8-week - 24 hour after	1.5313	0.223	1.085	1.978	49.00	6.873	<.001
Time2	Before - 24 hour after	-0.9837	0.223	-1.430	-0.537	49.00	-4.415	<.001
Time3	Immediately after - 24 hour after	-0.2587	0.223	-0.705	0.188	49.00	-1.161	0.251
Side1	Right - Left	-0.1654	0.158	-0.481	0.150	49.00	-1.050	0.299
Time1 ✻ Side1	(8-week - 24 hour after) ✻ (Right - Left)	-0.0609	0.446	-0.954	0.833	49.00	-0.137	0.892
Time2 ✻ Side1	(Before - 24 hour after) ✻ (Right - Left)	-0.2484	0.446	-1.142	0.645	49.00	-0.557	0.580
Time3 ✻ Side1	(Immediately after - 24 hour after) ✻ (Right - Left)	0.0891	0.446	-0.804	0.983	49.00	0.200	0.842

Random Components

Groups	Name	Variance	SD	ICC
**Horses**	(Intercept)	1.898	1.378	0.827
**Residual**		0.397	0.630	

Note. Number of Obs: 64 , Number of groups: Horses 8

Post Hoc comparison: Time

Comparison							
**Time**	**vs**	**Time**	**Difference**	**SE**	**t**	**df**	$p_{bonferroni}$
24 hour after	-	8-week	-1.531	0.223	-6.87	49.0	<.001
24 hour after	-	Before	0.984	0.223	4.41	49.0	<.001
24 hour after	-	Immediately after	0.259	0.223	1.16	49.0	1.000
8-week	-	Before	2.515	0.223	11.29	49.0	<.001
8-week	-	Immediately after	1.790	0.223	8.03	49.0	<.001
Before	-	Immediately after	-0.725	0.223	-3.25	49.0	0.012

Post Hoc comparison: Time ✻ Side

Comparison									
**Time**	**Side**	**vs**	**Time**	**Side**	**Difference**	**SE**	**t**	**df**	$p_{bonferroni}$
24 hour after	Left	-	24 hour after	Right	0.1104	0.315	0.3503	49.0	1.000
24 hour after	Left	-	8-week	Left	-1.5617	0.315	-4.9562	49.0	<.001
24 hour after	Left	-	8-week	Right	-1.3905	0.315	-4.4128	49.0	0.002
24 hour after	Left	-	Before	Left	0.8595	0.315	2.7276	49.0	0.247
24 hour after	Left	-	Before	Right	1.2182	0.315	3.8661	49.0	0.009
24 hour after	Left	-	Immediately after	Left	0.3033	0.315	0.9624	49.0	1.000
24 hour after	Left	-	Immediately after	Right	0.3245	0.315	1.0298	49.0	1.000

Post Hoc comparison: Time ✻ Side

Comparison									
**Time**	**Side**	**vs**	**Time**	**Side**	**Difference**	**SE**	**t**	**df**	$p_{bonferroni}$
24 hour after	Right	-	8-week	Left	-1.6721	0.315	-5.3065	49.0	<.001
24 hour after	Right	-	8-week	Right	-1.5009	0.315	-4.7631	49.0	<.001
24 hour after	Right	-	Before	Left	0.7491	0.315	2.3774	49.0	0.599
24 hour after	Right	-	Before	Right	1.1079	0.315	3.5159	49.0	0.027
24 hour after	Right	-	Immediately after	Left	0.1929	0.315	0.6121	49.0	1.000
24 hour after	Right	-	Immediately after	Right	0.2141	0.315	0.6795	49.0	1.000
8-week	Left	-	8-week	Right	0.1712	0.315	0.5435	49.0	1.000
8-week	Left	-	Before	Left	2.4213	0.315	7.6839	49.0	<.001
8-week	Left	-	Before	Right	2.7800	0.315	8.8224	49.0	<.001
8-week	Left	-	Immediately after	Left	1.8650	0.315	5.9186	49.0	<.001
8-week	Left	-	Immediately after	Right	1.8862	0.315	5.9861	49.0	<.001
8-week	Right	-	Before	Left	2.2500	0.315	7.1404	49.0	<.001
8-week	Right	-	Before	Right	2.6088	0.315	8.2789	49.0	<.001
8-week	Right	-	Immediately after	Left	1.6938	0.315	5.3752	49.0	<.001
8-week	Right	-	Immediately after	Right	1.7150	0.315	5.4426	49.0	<.001
Before	Left	-	Before	Right	0.3587	0.315	1.1385	49.0	1.000
Before	Left	-	Immediately after	Left	-0.5562	0.315	-1.7653	49.0	1.000
Before	Left	-	Immediately after	Right	-0.5350	0.315	-1.6978	49.0	1.000
Before	Right	-	Immediately after	Left	-0.9150	0.315	-2.9038	49.0	0.154

Post Hoc comparison: Time ✻ Side

Comparison									
Time	Side	vs	Time	Side	Difference	SE	t	df	$p_{bonferroni}$
Before	Right	-	Immediately after	Right	-0.8938	0.315	-2.8363	49.0	0.185
Immediately after	Left	-	Immediately after	Right	0.0212	0.315	0.0674	49.0	1.000

T16

Model Fit

Type	R^2	df	LRT X^2	p
Conditional	0.876	8	104.728	<.001
Marginal	0.189	7	60.877	<.001

Fixed Effects Omnibus Tests

	F	df	df (res)	p
**Time**	31.8809	3	49.0	<.001
**Side**	0.0247	1	49.0	0.876
**Time ✻ Side**	0.1644	3	49.0	0.920

Parameter Estimates (Fixed coefficients)

				95% Confidence Intervals				
Names	Effect	Estimate	SE	Lower	Upper	df	t	p
(Intercept)	(Intercept)	7.1755	0.449	6.275	8.0762	7.00	15.971	<.001
Time1	8-week - 24 hour after	0.8550	0.188	0.477	1.2328	49.00	4.538	<.001
Time2	Before - 24 hour after	-0.9194	0.188	-1.297	-0.5416	49.00	-4.879	<.001
Time3	Immediately after - 24 hour after	-0.4262	0.188	-0.804	-0.0485	49.00	-2.262	0.028
Side1	Right - Left	0.0209	0.133	-0.246	0.2881	49.00	0.157	0.876

Parameter Estimates (Fixed coefficients)

Names	Effect	Estimate	SE	95% Confidence Intervals Lower	95% Confidence Intervals Upper	df	t	p
Time1 ✻ Side1	(8-week - 24 hour after) ✻ (Right - Left)	0.1125	0.377	-0.643	0.8680	49.00	0.299	0.767
Time2 ✻ Side1	(Before - 24 hour after) ✻ (Right - Left)	0.1037	0.377	-0.652	0.8593	49.00	0.275	0.784
Time3 ✻ Side1	(Immediately after - 24 hour after) ✻ (Right - Left)	0.2625	0.377	-0.493	1.0180	49.00	0.697	0.489

Random Components

Groups	Name	Variance	SD	ICC
**Horses**	(Intercept)	1.579	1.257	0.848
**Residual**		0.284	0.533	

Note. Number of Obs: 64 , Number of groups: Horses 8

Post Hoc comparison: Time

Comparison Time	vs	Time	Difference	SE	t	df	$p_{bonferroni}$
24 hour after	-	8-week	-0.855	0.188	-4.54	49.0	<.001
24 hour after	-	Before	0.919	0.188	4.88	49.0	<.001
24 hour after	-	Immediately after	0.426	0.188	2.26	49.0	0.169
8-week	-	Before	1.774	0.188	9.42	49.0	<.001
8-week	-	Immediately after	1.281	0.188	6.80	49.0	<.001
Before	-	Immediately after	-0.493	0.188	-2.62	49.0	0.071

Post Hoc comparison: Side

Comparison							
**Side**	**vs**	**Side**	**Difference**	**SE**	**t**	**df**	**$p_{bonferroni}$**
Left	-	Right	-0.0209	0.133	-0.157	49.0	0.876

Post Hoc comparison: Time ✻ Side

Comparison									
**Time**	**Side**	**vs**	**Time**	**Side**	**Difference**	**SE**	**t**	**df**	**$p_{bonferroni}$**
24 hour after	Left	-	24 hour after	Right	0.09875	0.266	0.3706	49.0	1.000
24 hour after	Left	-	8-week	Left	-0.79875	0.266	-2.9975	49.0	0.119
24 hour after	Left	-	8-week	Right	-0.81250	0.266	-3.0491	49.0	0.103
24 hour after	Left	-	Before	Left	0.97125	0.266	3.6449	49.0	0.018
24 hour after	Left	-	Before	Right	0.96625	0.266	3.6261	49.0	0.019
24 hour after	Left	-	Immediately after	Left	0.55750	0.266	2.0922	49.0	1.000
24 hour after	Left	-	Immediately after	Right	0.39375	0.266	1.4777	49.0	1.000
24 hour after	Right	-	8-week	Left	-0.89750	0.266	-3.3681	49.0	0.041
24 hour after	Right	-	8-week	Right	-0.91125	0.266	-3.4197	49.0	0.036
24 hour after	Right	-	Before	Left	0.87250	0.266	3.2743	49.0	0.055
24 hour after	Right	-	Before	Right	0.86750	0.266	3.2555	49.0	0.058
24 hour after	Right	-	Immediately after	Left	0.45875	0.266	1.7216	49.0	1.000
24 hour after	Right	-	Immediately after	Right	0.29500	0.266	1.1071	49.0	1.000
8-week	Left	-	8-week	Right	-0.01375	0.266	-0.0516	49.0	1.000
8-week	Left	-	Before	Left	1.77000	0.266	6.6424	49.0	<.001

Post Hoc comparison: Time ✻ Side

Comparison									
Time	Side	vs	Time	Side	Difference	SE	t	df	$p_{bonferroni}$
8-week	Left	-	Before	Right	1.76500	0.266	6.6237	49.0	<.001
8-week	Left	-	Immediately after	Left	1.35625	0.266	5.0897	49.0	<.001
8-week	Left	-	Immediately after	Right	1.19250	0.266	4.4752	49.0	0.001
8-week	Right	-	Before	Left	1.78375	0.266	6.6940	49.0	<.001
8-week	Right	-	Before	Right	1.77875	0.266	6.6753	49.0	<.001
8-week	Right	-	Immediately after	Left	1.37000	0.266	5.1413	49.0	<.001
8-week	Right	-	Immediately after	Right	1.20625	0.266	4.5268	49.0	0.001
Before	Left	-	Before	Right	-0.00500	0.266	-0.0188	49.0	1.000
Before	Left	-	Immediately after	Left	-0.41375	0.266	-1.5527	49.0	1.000
Before	Left	-	Immediately after	Right	-0.57750	0.266	-2.1672	49.0	0.983
Before	Right	-	Immediately after	Left	-0.40875	0.266	-1.5340	49.0	1.000
Before	Right	-	Immediately after	Right	-0.57250	0.266	-2.1485	49.0	1.000
Immediately after	Left	-	Immediately after	Right	-0.16375	0.266	-0.6145	49.0	1.000

T14

Model Fit

Type	R^2	df	LRT X^2	p
Conditional	0.933	8	138.244	<.001
Marginal	0.163	7	79.652	<.001

Fixed Effects Omnibus Tests

	F	df	df (res)	p
**Time**	49.6096	3	49.0	<.001
**Side**	4.9024	1	49.0	0.032
**Time ✻ Side**	0.0825	3	49.0	0.969

Parameter Estimates (Fixed coefficients)

				95% Confidence Intervals				
**Names**	**Effect**	**Estimate**	**SE**	**Lower**	**Upper**	**df**	**t**	**p**
(Intercept)	(Intercept)	6.74562	0.513	5.7179	7.773	7.00	13.1595	<.001
Time1	8-week - 24 hour after	0.71188	0.150	0.4109	1.013	49.00	4.7427	<.001
Time2	Before - 24 hour after	-1.03500	0.150	-1.3359	-0.734	49.00	-6.8954	<.001
Time3	Immediately after - 24 hour after	-0.52937	0.150	-0.8303	-0.228	49.00	-3.5268	<.001
Side1	Right - Left	0.23500	0.106	0.0222	0.448	49.00	2.2141	0.032
Time1 ✻ Side1	(8-week - 24 hour after) ✻ (Right - Left)	-0.12375	0.300	-0.7256	0.478	49.00	-0.4122	0.682
Time2 ✻ Side1	(Before - 24 hour after) ✻ (Right - Left)	0.01000	0.300	-0.5919	0.612	49.00	0.0333	0.974
Time3 ✻ Side1	(Immediately after - 24 hour after) ✻ (Right - Left)	-0.04625	0.300	-0.6481	0.556	49.00	-0.1541	0.878

Random Components

Groups	Name	Variance	SD	ICC
**Horses**	(Intercept)	2.080	1.442	0.920
**Residual**		0.180	0.425	

Random Components

Groups	Name	Variance	SD	ICC

Note. Number of Obs: 64 , Number of groups: Horses 8

Post Hoc comparison: Time

Comparison							
Time	vs	Time	Difference	SE	t	df	$p_{bonferroni}$
24 hour after	-	8-week	-0.712	0.150	-4.74	49.0	<.001
24 hour after	-	Before	1.035	0.150	6.90	49.0	<.001
24 hour after	-	Immediately after	0.529	0.150	3.53	49.0	0.006
8-week	-	Before	1.747	0.150	11.64	49.0	<.001
8-week	-	Immediately after	1.241	0.150	8.27	49.0	<.001
Before	-	Immediately after	-0.506	0.150	-3.37	49.0	0.009

Post Hoc comparison: Side

Comparison							
Side	vs	Side	Difference	SE	t	df	$p_{bonferroni}$
Left	-	Right	-0.235	0.106	-2.21	49.0	0.032

Post Hoc comparison: Time ✻ Side

Comparison									
Time	Side	vs	Time	Side	Difference	SE	t	df	$p_{bonferroni}$
24 hour after	Left	-	24 hour after	Right	-0.275	0.212	-1.296	49.0	1.000
24 hour after	Left	-	8-week	Left	-0.774	0.212	-3.645	49.0	0.018
24 hour after	Left	-	8-week	Right	-0.925	0.212	-4.358	49.0	0.002
24 hour after	Left	-	Before	Left	1.040	0.212	4.899	49.0	<.001

Post Hoc comparison: Time ✻ Side

Comparison									
Time	Side	vs	Time	Side	Difference	SE	t	df	$p_{bonferroni}$
24 hour after	Left	-	Before	Right	0.755	0.212	3.557	49.0	0.024
24 hour after	Left	-	Immediately after	Left	0.506	0.212	2.385	49.0	0.588
24 hour after	Left	-	Immediately after	Right	0.277	0.212	1.307	49.0	1.000
24 hour after	Right	-	8-week	Left	-0.499	0.212	-2.350	49.0	0.640
24 hour after	Right	-	8-week	Right	-0.650	0.212	-3.062	49.0	0.100
24 hour after	Right	-	Before	Left	1.315	0.212	6.195	49.0	<.001
24 hour after	Right	-	Before	Right	1.030	0.212	4.852	49.0	<.001
24 hour after	Right	-	Immediately after	Left	0.781	0.212	3.680	49.0	0.016
24 hour after	Right	-	Immediately after	Right	0.552	0.212	2.603	49.0	0.342
8-week	Left	-	8-week	Right	-0.151	0.212	-0.713	49.0	1.000
8-week	Left	-	Before	Left	1.814	0.212	8.544	49.0	<.001
8-week	Left	-	Before	Right	1.529	0.212	7.202	49.0	<.001
8-week	Left	-	Immediately after	Left	1.280	0.212	6.030	49.0	<.001
8-week	Left	-	Immediately after	Right	1.051	0.212	4.952	49.0	<.001
8-week	Right	-	Before	Left	1.965	0.212	9.257	49.0	<.001
8-week	Right	-	Before	Right	1.680	0.212	7.914	49.0	<.001
8-week	Right	-	Immediately after	Left	1.431	0.212	6.743	49.0	<.001
8-week	Right	-	Immediately after	Right	1.202	0.212	5.665	49.0	<.001
Before	Left	-	Before	Right	-0.285	0.212	-1.343	49.0	1.000

Post Hoc comparison: Time ✻ Side

Comparison									
Time	Side	vs	Time	Side	Difference	SE	t	df	$p_{bonferroni}$
Before	Left	-	Immediately after	Left	-0.534	0.212	-2.514	49.0	0.427
Before	Left	-	Immediately after	Right	-0.762	0.212	-3.592	49.0	0.021
Before	Right	-	Immediately after	Left	-0.249	0.212	-1.172	49.0	1.000
Before	Right	-	Immediately after	Right	-0.478	0.212	-2.249	49.0	0.812
Immediately after	Left	-	Immediately after	Right	-0.229	0.212	-1.078	49.0	1.000

T12

Model Fit

Type	R^2	df	LRT X^2	p
Conditional	0.915	8	122.865	<.001
Marginal	0.063	7	37.407	<.001

Fixed Effects Omnibus Tests

	F	df	df (res)	p
**Time**	12.1073	3	49.0	<.001
**Side**	9.9618	1	49.0	0.003
**Time ✻ Side**	0.0592	3	49.0	0.981

Parameter Estimates (Fixed coefficients)

Names	Effect	Estimate	SE	95% Confidence Intervals Lower	95% Confidence Intervals Upper	df	t	p
(Intercept)	(Intercept)	4.9300	0.612	3.704	6.1562	7.00	8.0611	<.001
Time1	8-week - 24 hour after	0.6743	0.192	0.289	1.0601	49.00	3.5047	<.001
Time2	Before - 24 hour after	-0.4444	0.192	-0.830	-0.0587	49.00	-2.3099	0.025
Time3	Immediately after - 24 hour after	-0.1476	0.192	-0.533	0.2382	49.00	-0.7669	0.447
Side1	Right - Left	0.4294	0.136	0.157	0.7022	49.00	3.1562	0.003
Time1 ✻ Side1	(8-week - 24 hour after) ✻ (Right - Left)	-0.0289	0.385	-0.800	0.7426	49.00	-0.0750	0.940
Time2 ✻ Side1	(Before - 24 hour after) ✻ (Right - Left)	-0.1514	0.385	-0.923	0.6201	49.00	-0.3934	0.696
Time3 ✻ Side1	(Immediately after - 24 hour after) ✻ (Right - Left)	-0.0776	0.385	-0.849	0.6939	49.00	-0.2017	0.841

Random Components

Groups	Name	Variance	SD	ICC
**Horses**	(Intercept)	2.955	1.719	0.909
**Residual**		0.296	0.544	

Note. Number of Obs: 64 , Number of groups: Horses 8

Post Hoc comparison: Time

Comparison: Time	vs	Time	Difference	SE	t	df	$p_{bonferroni}$
24 hour after	-	8-week	-0.674	0.192	-3.505	49.0	0.006
24 hour after	-	Before	0.444	0.192	2.310	49.0	0.151
24 hour after	-	Immediately after	0.148	0.192	0.767	49.0	1.000
8-week	-	Before	1.119	0.192	5.815	49.0	<.001
8-week	-	Immediately after	0.822	0.192	4.272	49.0	<.001

Post Hoc comparison: Time

Comparison							
Time	vs	Time	Difference	SE	t	df	$p_{bonferroni}$
Before	-	Immediately after	-0.297	0.192	-1.543	49.0	0.776

Post Hoc comparison: Side

Comparison							
Side	vs	Side	Difference	SE	t	df	$p_{bonferroni}$
Left	-	Right	-0.429	0.136	-3.16	49.0	0.003

Post Hoc comparison: Time ✻ Side

Comparison									
Time	Side	vs	Time	Side	Difference	SE	t	df	$p_{bonferroni}$
24 hour after	Left	-	24 hour after	Right	-0.4939	0.272	-1.8150	49.0	1.000
24 hour after	Left	-	8-week	Left	-0.6888	0.272	-2.5312	49.0	0.410
24 hour after	Left	-	8-week	Right	-1.1538	0.272	-4.2402	49.0	0.003
24 hour after	Left	-	Before	Left	0.3688	0.272	1.3552	49.0	1.000
24 hour after	Left	-	Before	Right	0.0263	0.272	0.0965	49.0	1.000
24 hour after	Left	-	Immediately after	Left	0.1087	0.272	0.3997	49.0	1.000
24 hour after	Left	-	Immediately after	Right	-0.3075	0.272	-1.1301	49.0	1.000
24 hour after	Right	-	8-week	Left	-0.1949	0.272	-0.7162	49.0	1.000
24 hour after	Right	-	8-week	Right	-0.6599	0.272	-2.4251	49.0	0.533
24 hour after	Right	-	Before	Left	0.8626	0.272	3.1702	49.0	0.074

Post Hoc comparison: Time ✻ Side

Comparison									
Time	Side	vs	Time	Side	Difference	SE	t	df	$p_{bonferroni}$
24 hour after	Right	-	Before	Right	0.5201	0.272	1.9115	49.0	1.000
24 hour after	Right	-	Immediately after	Left	0.6026	0.272	2.2147	49.0	0.881
24 hour after	Right	-	Immediately after	Right	0.1864	0.272	0.6850	49.0	1.000
8-week	Left	-	8-week	Right	-0.4650	0.272	-1.7089	49.0	1.000
8-week	Left	-	Before	Left	1.0575	0.272	3.8864	49.0	0.009
8-week	Left	-	Before	Right	0.7150	0.272	2.6277	49.0	0.320
8-week	Left	-	Immediately after	Left	0.7975	0.272	2.9309	49.0	0.143
8-week	Left	-	Immediately after	Right	0.3813	0.272	1.4011	49.0	1.000
8-week	Right	-	Before	Left	1.5225	0.272	5.5954	49.0	<.001
8-week	Right	-	Before	Right	1.1800	0.272	4.3366	49.0	0.002
8-week	Right	-	Immediately after	Left	1.2625	0.272	4.6398	49.0	<.001
8-week	Right	-	Immediately after	Right	0.8462	0.272	3.1101	49.0	0.087
Before	Left	-	Before	Right	-0.3425	0.272	-1.2587	49.0	1.000
Before	Left	-	Immediately after	Left	-0.2600	0.272	-0.9555	49.0	1.000
Before	Left	-	Immediately after	Right	-0.6763	0.272	-2.4853	49.0	0.459
Before	Right	-	Immediately after	Left	0.0825	0.272	0.3032	49.0	1.000
Before	Right	-	Immediately after	Right	-0.3338	0.272	-1.2266	49.0	1.000
Immediately after	Left	-	Immediately after	Right	-0.4163	0.272	-1.5298	49.0	1.000

T10

Model Fit

Type	R^2	df	LRT X^2	p
Conditional	0.880	8	104.607	<.001
Marginal	0.103	7	41.609	<.001

Fixed Effects Omnibus Tests

	F	df	df (res)	p
**Time**	13.291	3	49.0	<.001
**Side**	12.639	1	49.0	<.001
**Time ✻ Side**	0.463	3	49.0	0.710

Parameter Estimates (Fixed coefficients)

				95% Confidence Intervals				
**Names**	**Effect**	**Estimate**	**SE**	**Lower**	**Upper**	**df**	**t**	**p**
(Intercept)	(Intercept)	3.4636	0.485	2.490	4.437	7.00	7.1345	<.001
Time1	8-week - 24 hour after	0.6389	0.189	0.259	1.018	49.00	3.3747	0.001
Time2	Before - 24 hour after	- 0.5053	0.189	- 0.885	- 0.126	49.00	- 2.6692	0.010
Time3	Immediately after - 24 hour after	- 0.2311	0.189	- 0.611	0.148	49.00	- 1.2208	0.228
Side1	Right - Left	0.4759	0.134	0.208	0.744	49.00	3.5551	<.001
Time1 ✻ Side1	(8-week - 24 hour after) ✻ (Right - Left)	- 0.1752	0.379	- 0.934	0.584	49.00	- 0.4629	0.646
Time2 ✻ Side1	(Before - 24 hour after) ✻ (Right - Left)	0.0156	0.379	- 0.743	0.775	49.00	0.0413	0.967
Time3 ✻ Side1	(Immediately after - 24 hour after) ✻ (Right - Left)	0.2673	0.379	- 0.492	1.026	49.00	0.7058	0.484

Random Components

Groups	Name	Variance	SD	ICC
**Horses**	(Intercept)	1.850	1.360	0.866
**Residual**		0.287	0.535	

Note. Number of Obs: 64 , Number of groups: Horses 8

Post Hoc comparison: Time

Comparison							
**Time**	**vs**	**Time**	**Difference**	**SE**	**t**	**df**	$p_{bonferroni}$
24 hour after	-	8-week	-0.639	0.189	-3.37	49.0	0.009
24 hour after	-	Before	0.505	0.189	2.67	49.0	0.062
24 hour after	-	Immediately after	0.231	0.189	1.22	49.0	1.000
8-week	-	Before	1.144	0.189	6.04	49.0	<.001
8-week	-	Immediately after	0.870	0.189	4.60	49.0	<.001
Before	-	Immediately after	-0.274	0.189	-1.45	49.0	0.923

Post Hoc comparison: Side

Comparison							
**Side**	**vs**	**Side**	**Difference**	**SE**	**t**	**df**	$p_{bonferroni}$
Left	-	Right	-0.476	0.134	-3.56	49.0	<.001

Post Hoc comparison: Time ✻ Side

Comparison									
**Time**	**Side**	**vs**	**Time**	**Side**	**Difference**	**SE**	**t**	**df**	$p_{bonferroni}$
24 hour after	Left	-	24 hour after	Right	-0.4490	0.268	-1.677	49.0	1.000
24 hour after	Left	-	8-week	Left	-0.7265	0.268	-2.714	49.0	0.257
24 hour after	Left	-	8-week	Right	-1.0003	0.268	-3.736	49.0	0.014

Post Hoc comparison: Time ✻ Side

Comparison									
**Time**	**Side**	**vs**	**Time**	**Side**	**Difference**	**SE**	**t**	**df**	**$p_{bonferroni}$**
24 hour after	Left	-	Before	Left	0.5131	0.268	1.917	49.0	1.000
24 hour after	Left	-	Before	Right	0.0485	0.268	0.181	49.0	1.000
24 hour after	Left	-	Immediately after	Left	0.3647	0.268	1.362	49.0	1.000
24 hour after	Left	-	Immediately after	Right	-0.3515	0.268	-1.313	49.0	1.000
24 hour after	Right	-	8-week	Left	-0.2775	0.268	-1.036	49.0	1.000
24 hour after	Right	-	8-week	Right	-0.5513	0.268	-2.059	49.0	1.000
24 hour after	Right	-	Before	Left	0.9621	0.268	3.594	49.0	0.021
24 hour after	Right	-	Before	Right	0.4975	0.268	1.858	49.0	1.000
24 hour after	Right	-	Immediately after	Left	0.8137	0.268	3.039	49.0	0.106
24 hour after	Right	-	Immediately after	Right	0.0975	0.268	0.364	49.0	1.000
8-week	Left	-	8-week	Right	-0.2738	0.268	-1.022	49.0	1.000
8-week	Left	-	Before	Left	1.2396	0.268	4.630	49.0	<.001
8-week	Left	-	Before	Right	0.7750	0.268	2.895	49.0	0.158
8-week	Left	-	Immediately after	Left	1.0912	0.268	4.076	49.0	0.005
8-week	Left	-	Immediately after	Right	0.3750	0.268	1.401	49.0	1.000
8-week	Right	-	Before	Left	1.5134	0.268	5.653	49.0	<.001
8-week	Right	-	Before	Right	1.0488	0.268	3.917	49.0	0.008
8-week	Right	-	Immediately after	Left	1.3650	0.268	5.098	49.0	<.001
8-week	Right	-	Immediately after	Right	0.6488	0.268	2.423	49.0	0.536

Post Hoc comparison: Time ✻ Side

Comparison									
Time	Side	vs	Time	Side	Difference	SE	t	df	$p_{bonferroni}$
Before	Left	-	Before	Right	-0.4646	0.268	-1.735	49.0	1.000
Before	Left	-	Immediately after	Left	-0.1484	0.268	-0.554	49.0	1.000
Before	Left	-	Immediately after	Right	-0.8646	0.268	-3.229	49.0	0.062
Before	Right	-	Immediately after	Left	0.3163	0.268	1.181	49.0	1.000
Before	Right	-	Immediately after	Right	-0.4000	0.268	-1.494	49.0	1.000
Immediately after	Left	-	Immediately after	Right	-0.7163	0.268	-2.675	49.0	0.283

L2

Model Fit

Type	R^2	df	LRT X^2	p
Conditional	0.805	8	80.404	<.001
Marginal	0.210	7	48.703	<.001

Fixed Effects Omnibus Tests

	F	df	df (res)	p
**Time**	19.027	3	49.0	<.001
**Side**	9.126	1	49.0	0.004
**Time ✻ Side**	0.530	3	49.0	0.664

Parameter Estimates (Fixed coefficients)

Names	Effect	Estimate	SE	95% Confidence Intervals Lower	Upper	df	t	p
(Intercept)	(Intercept)	5.54620	0.405	4.734	6.359	7.00	13.6834	<.001
Time1	8-week - 24 hour after	0.78956	0.227	0.334	1.245	49.00	3.4724	0.001
Time2	Before - 24 hour after	-0.89294	0.227	-1.349	-0.437	49.00	-3.9271	<.001
Time3	Immediately after - 24 hour after	-0.31356	0.227	-0.769	0.142	49.00	-1.3790	0.174
Side1	Right - Left	0.48572	0.161	0.163	0.808	49.00	3.0210	0.004
Time1 ✻ Side1	(8-week - 24 hour after) ✻ (Right - Left)	-0.43663	0.455	-1.348	0.475	49.00	-0.9601	0.342
Time2 ✻ Side1	(Before - 24 hour after) ✻ (Right - Left)	-0.00913	0.455	-0.921	0.903	49.00	-0.0201	0.984
Time3 ✻ Side1	(Immediately after - 24 hour after) ✻ (Right - Left)	0.08212	0.455	-0.830	0.994	49.00	0.1806	0.857

Random Components

Groups	Name	Variance	SD	ICC
**Horses**	(Intercept)	1.263	1.124	0.753
**Residual**		0.414	0.643	

Note. Number of Obs: 64 , Number of groups: Horses 8

Post Hoc comparison: Time

Comparison Time	vs	Time	Difference	SE	t	df	$p_{bonferroni}$
24 hour after	-	8-week	-0.790	0.227	-3.47	49.0	0.007
24 hour after	-	Before	0.893	0.227	3.93	49.0	0.002
24 hour after	-	Immediately after	0.314	0.227	1.38	49.0	1.000

Post Hoc comparison: Time

Comparison							
Time	vs	Time	Difference	SE	t	df	$p_{bonferroni}$
8-week	-	Before	1.682	0.227	7.40	49.0	<.001
8-week	-	Immediately after	1.103	0.227	4.85	49.0	<.001
Before	-	Immediately after	-0.579	0.227	-2.55	49.0	0.084

Post Hoc comparison: Side

Comparison							
Side	vs	Side	Difference	SE	t	df	$p_{bonferroni}$
Left	-	Right	-0.486	0.161	-3.02	49.0	0.004

Post Hoc comparison: Time ✻ Side

Comparison									
Time	Side	vs	Time	Side	Difference	SE	t	df	$p_{bonferroni}$
24 hour after	Left	-	24 hour after	Right	-0.5766	0.322	-1.793	49.0	1.000
24 hour after	Left	-	8-week	Left	-1.0079	0.322	-3.134	49.0	0.081
24 hour after	Left	-	8-week	Right	-1.1479	0.322	-3.570	49.0	0.023
24 hour after	Left	-	Before	Left	0.8884	0.322	2.763	49.0	0.225
24 hour after	Left	-	Before	Right	0.3209	0.322	0.998	49.0	1.000
24 hour after	Left	-	Immediately after	Left	0.3546	0.322	1.103	49.0	1.000
24 hour after	Left	-	Immediately after	Right	-0.3041	0.322	-0.946	49.0	1.000
24 hour after	Right	-	8-week	Left	-0.4313	0.322	-1.341	49.0	1.000
24 hour after	Right	-	8-week	Right	-0.5712	0.322	-1.776	49.0	1.000

Post Hoc comparison: Time ✻ Side

Comparison									
**Time**	**Side**	**vs**	**Time**	**Side**	**Difference**	**SE**	**t**	**df**	**$p_{bonferroni}$**
24 hour after	Right	-	Before	Left	1.4650	0.322	4.556	49.0	<.001
24 hour after	Right	-	Before	Right	0.8975	0.322	2.791	49.0	0.209
24 hour after	Right	-	Immediately after	Left	0.9312	0.322	2.896	49.0	0.158
24 hour after	Right	-	Immediately after	Right	0.2725	0.322	0.847	49.0	1.000
8-week	Left	-	8-week	Right	-0.1400	0.322	-0.435	49.0	1.000
8-week	Left	-	Before	Left	1.8962	0.322	5.897	49.0	<.001
8-week	Left	-	Before	Right	1.3288	0.322	4.132	49.0	0.004
8-week	Left	-	Immediately after	Left	1.3625	0.322	4.237	49.0	0.003
8-week	Left	-	Immediately after	Right	0.7038	0.322	2.189	49.0	0.936
8-week	Right	-	Before	Left	2.0362	0.322	6.332	49.0	<.001
8-week	Right	-	Before	Right	1.4688	0.322	4.568	49.0	<.001
8-week	Right	-	Immediately after	Left	1.5025	0.322	4.672	49.0	<.001
8-week	Right	-	Immediately after	Right	0.8438	0.322	2.624	49.0	0.324
Before	Left	-	Before	Right	-0.5675	0.322	-1.765	49.0	1.000
Before	Left	-	Immediately after	Left	-0.5337	0.322	-1.660	49.0	1.000
Before	Left	-	Immediately after	Right	-1.1925	0.322	-3.708	49.0	0.015
Before	Right	-	Immediately after	Left	0.0337	0.322	0.105	49.0	1.000
Before	Right	-	Immediately after	Right	-0.6250	0.322	-1.944	49.0	1.000
Immediately after	Left	-	Immediately after	Right	-0.6587	0.322	-2.049	49.0	1.000

L5

Model Fit

Type	R^2	df	LRT X^2	p
Conditional	0.912	8	125.269	<.001
Marginal	0.296	7	93.448	<.001

Fixed Effects Omnibus Tests

	F	df	df (res)	p
**Time**	68.894	3	49.0	<.001
**Side**	2.335	1	49.0	0.133
**Time ✻ Side**	0.555	3	49.0	0.648

Parameter Estimates (Fixed coefficients)

				95% Confidence Intervals				
**Names**	**Effect**	**Estimate**	**SE**	**Lower**	**Upper**	**df**	**t**	**p**
(Intercept)	(Intercept)	5.4984	0.3681	4.7604	6.237	7.00	14.936	<.001
Time1	8-week - 24 hour after	1.0169	0.1382	0.7397	1.294	49.00	7.356	<.001
Time2	Before - 24 hour after	-0.8806	0.1382	-1.1578	-0.603	49.00	-6.371	<.001
Time3	Immediately after - 24 hour after	-0.4325	0.1382	-0.7096	-0.155	49.00	-3.129	0.003
Side1	Right - Left	0.1494	0.0977	-0.0466	0.345	49.00	1.528	0.133
Time1 ✻ Side1	(8-week - 24 hour after) ✻ (Right - Left)	0.0638	0.2765	-0.4905	0.618	49.00	0.231	0.819
Time2 ✻ Side1	(Before - 24 hour after) ✻ (Right - Left)	-0.0363	0.2765	-0.5905	0.518	49.00	-0.131	0.896

Parameter Estimates (Fixed coefficients)

Names	Effect	Estimate	SE	95% Confidence Intervals Lower	95% Confidence Intervals Upper	df	t	p
Time3 ✻ Side1	(Immediately after - 24 hour after) ✻ (Right - Left)	-0.2700	0.2765	-0.8243	0.284	49.00	-0.977	0.334

Random Components

Groups	Name	Variance	SD	ICC
**Horses**	(Intercept)	1.065	1.032	0.874
**Residual**		0.153	0.391	

Note. Number of Obs: 64 , Number of groups: Horses 8

Post Hoc comparison: Time

Comparison							
Time	vs	Time	Difference	SE	t	df	$p_{bonferroni}$
24 hour after	-	8-week	-1.017	0.138	-7.36	49.0	<.001
24 hour after	-	Before	0.881	0.138	6.37	49.0	<.001
24 hour after	-	Immediately after	0.433	0.138	3.13	49.0	0.018
8-week	-	Before	1.897	0.138	13.73	49.0	<.001
8-week	-	Immediately after	1.449	0.138	10.49	49.0	<.001
Before	-	Immediately after	-0.448	0.138	-3.24	49.0	0.013

Post Hoc comparison: Side

Comparison							
Side	vs	Side	Difference	SE	t	df	$p_{bonferroni}$
Left	-	Right	-0.149	0.0977	-1.53	49.0	0.133

Post Hoc comparison: Time ✻ Side

Comparison									
**Time**	**Side**	**vs**	**Time**	**Side**	**Difference**	**SE**	**t**	**df**	**$p_{bonferroni}$**
24 hour after	Left	-	24 hour after	Right	-0.2100	0.195	-1.074	49.0	1.000
24 hour after	Left	-	8-week	Left	-0.9850	0.195	-5.039	49.0	<.001
24 hour after	Left	-	8-week	Right	-1.2588	0.195	-6.439	49.0	<.001
24 hour after	Left	-	Before	Left	0.8625	0.195	4.412	49.0	0.002
24 hour after	Left	-	Before	Right	0.6887	0.195	3.523	49.0	0.026
24 hour after	Left	-	Immediately after	Left	0.2975	0.195	1.522	49.0	1.000
24 hour after	Left	-	Immediately after	Right	0.3575	0.195	1.829	49.0	1.000
24 hour after	Right	-	8-week	Left	-0.7750	0.195	-3.964	49.0	0.007
24 hour after	Right	-	8-week	Right	-1.0487	0.195	-5.365	49.0	<.001
24 hour after	Right	-	Before	Left	1.0725	0.195	5.486	49.0	<.001
24 hour after	Right	-	Before	Right	0.8988	0.195	4.597	49.0	<.001
24 hour after	Right	-	Immediately after	Left	0.5075	0.195	2.596	49.0	0.348
24 hour after	Right	-	Immediately after	Right	0.5675	0.195	2.903	49.0	0.155
8-week	Left	-	8-week	Right	-0.2738	0.195	-1.400	49.0	1.000
8-week	Left	-	Before	Left	1.8475	0.195	9.451	49.0	<.001
8-week	Left	-	Before	Right	1.6737	0.195	8.562	49.0	<.001
8-week	Left	-	Immediately after	Left	1.2825	0.195	6.560	49.0	<.001
8-week	Left	-	Immediately after	Right	1.3425	0.195	6.867	49.0	<.001
8-week	Right	-	Before	Left	2.1213	0.195	10.851	49.0	<.001
8-week	Right	-	Before	Right	1.9475	0.195	9.962	49.0	<.001
8-week	Right	-	Immediately after	Left	1.5563	0.195	7.961	49.0	<.001

Post Hoc comparison: Time ✻ Side

Comparison									
Time	Side	vs	Time	Side	Difference	SE	t	df	$p_{bonferroni}$
8-week	Right	-	Immediately after	Right	1.6163	0.195	8.268	49.0	<.001
Before	Left	-	Before	Right	-0.1737	0.195	-0.889	49.0	1.000
Before	Left	-	Immediately after	Left	-0.5650	0.195	-2.890	49.0	0.160
Before	Left	-	Immediately after	Right	-0.5050	0.195	-2.583	49.0	0.359
Before	Right	-	Immediately after	Left	-0.3912	0.195	-2.001	49.0	1.000
Before	Right	-	Immediately after	Right	-0.3313	0.195	-1.694	49.0	1.000
Immediately after	Left	-	Immediately after	Right	0.0600	0.195	0.307	49.0	1.000

AR W-T13

Model Fit

Type	R^2	df	LRT X^2	p
Conditional	0.620	4	22.224	<.001
Marginal	0.382	3	21.900	<.001

Fixed Effects Omnibus Tests

	F	df	df (res)	p
**Time**	10.4	3	21.0	<.001

Parameter Estimates (Fixed coefficients)

				95% Confidence Intervals				
Names	Effect	Estimate	SE	Lower	Upper	df	t	p
(Intercept)	(Intercept)	12.284	0.734	10.777	13.79	7.00	16.747	<.001
Time1	T2 - T1	1.463	1.108	-0.815	3.74	21.00	1.320	0.201
Time2	T3 - T1	4.800	1.108	2.523	7.08	21.00	4.333	<.001

Parameter Estimates (Fixed coefficients)

Names	Effect	Estimate	SE	95% Confidence Intervals Lower	Upper	df	t	p
Time3	T4 - T1	-0.975	1.108	-3.252	1.30	21.00	-0.880	0.389

Random Components

Groups	Name	Variance	SD	ICC
**Horses**	(Intercept)	3.08	1.75	0.385
**Residual**		4.91	2.22	

Note. Number of Obs: 32 , Number of groups: Horses 8

Post Hoc comparison: Time

Comparison Time	vs	Time	Difference	SE	t	df	p_{tukey}
T1	-	T2	-1.463	1.11	-1.320	21.0	0.561
T1	-	T3	-4.800	1.11	-4.333	21.0	0.002
T1	-	T4	0.975	1.11	0.880	21.0	0.815
T2	-	T3	-3.338	1.11	-3.013	21.0	0.031
T2	-	T4	2.438	1.11	2.200	21.0	0.156
T3	-	T4	5.775	1.11	5.213	21.0	<.001

AR T13T18

Model Fit

Type	R^2	df	LRT X^2	p
Conditional	0.325	4	12.608	0.013
Marginal	0.303	3	12.608	0.006

Fixed Effects Omnibus Tests

	F	df	df (res)	p
**Time**	4.63	3	21.0	0.012

Parameter Estimates (Fixed coefficients)

				95% Confidence Intervals				
**Names**	**Effect**	**Estimate**	**SE**	**Lower**	**Upper**	**df**	**t**	**p**
(Intercept)	(Intercept)	9.37	0.782	7.762	10.975	7.00	11.99	<.001
Time1	T2 - T1	2.31	2.081	-1.965	6.590	21.00	1.11	0.279
Time2	T3 - T1	3.88	2.081	-0.402	8.152	21.00	1.86	0.077
Time3	T4 - T1	-3.41	2.081	-7.690	0.865	21.00	-1.64	0.116

Random Components

Groups	Name	Variance	SD	ICC
**Horses**	(Intercept)	0.558	0.747	0.0312
**Residual**		17.318	4.162	

Note. Number of Obs: 32 , Number of groups: Horses 8

Post Hoc comparison: Time

Comparison					95% Confidence Intervals				
**Time**	**vs**	**Time**	**Difference**	**SE**	**Lower**	**Upper**	**t**	**df**	**p_{tukey}**
T1	-	T2	-2.31	2.08	-8.1123	3.49	-1.111	21.0	0.687
T1	-	T3	-3.88	2.08	-9.6748	1.92	-1.862	21.0	0.274
T1	-	T4	3.41	2.08	-2.3873	9.21	1.640	21.0	0.379
T2	-	T3	-1.56	2.08	-7.3623	4.24	-0.751	21.0	0.875
T2	-	T4	5.72	2.08	-0.0748	11.52	2.751	21.0	0.054
T3	-	T4	7.29	2.08	1.4877	13.09	3.502	21.0	0.011

AR T18L3

Model Fit

Type	R^2	df	LRT X^2	p
Conditional	0.371	4	12.559	0.014
Marginal	0.292	3	12.573	0.006

Fixed Effects Omnibus Tests

	F	df	df (res)	p
**Time**	4.79	3	21.0	0.011

Parameter Estimates (Fixed coefficients)

				95% Confidence Intervals				
**Names**	**Effect**	**Estimate**	**SE**	**Lower**	**Upper**	**df**	**t**	**p**
(Intercept)	(Intercept)	9.616	0.564	8.46	10.78	7.00	17.046	<.001
Time1	T2 - T1	-1.175	1.302	-3.85	1.50	21.00	-0.903	0.377
Time2	T3 - T1	0.137	1.302	-2.54	2.81	21.00	0.106	0.917
Time3	T4 - T1	-4.200	1.302	-6.88	-1.52	21.00	-3.227	0.004

Random Components

Groups	Name	Variance	SD	ICC
**Horses**	(Intercept)	0.852	0.923	0.112
**Residual**		6.776	2.603	

Note. Number of Obs: 32 , Number of groups: Horses 8

Post Hoc comparison: Time

Comparison							
**Time**	**vs**	**Time**	**Difference**	**SE**	**t**	**df**	**p_{tukey}**
T1	-	T2	1.175	1.30	0.903	21.0	0.803
T1	-	T3	-0.137	1.30	-0.106	21.0	1.000
T1	-	T4	4.200	1.30	3.227	21.0	0.019

Post Hoc comparison: Time

Comparison							
Time	vs	Time	Difference	SE	t	df	p_{tukey}
T2	-	T3	-1.312	1.30	-1.008	21.0	0.746
T2	-	T4	3.025	1.30	2.324	21.0	0.124
T3	-	T4	4.338	1.30	3.333	21.0	0.015

AR L3S

Model Fit

Type	R^2	df	LRT X^2	p
Conditional	0.330	4	7.822	0.098
Marginal	0.162	3	7.395	0.060

Fixed Effects Omnibus Tests

	F	df	df (res)	p
**Time**	2.51	3	21.0	0.087

Parameter Estimates (Fixed coefficients)

				95% Confidence Intervals				
Names	Effect	Estimate	SE	Lower	Upper	df	t	p
(Intercept)	(Intercept)	10.894	0.650	9.5571	12.23	7.00	16.753	<.001
Time1	T2 - T1	0.575	1.300	-2.0976	3.25	21.00	0.442	0.663
Time2	T3 - T1	2.763	1.300	0.0899	5.44	21.00	2.125	0.046
Time3	T4 - T1	2.787	1.300	0.1149	5.46	21.00	2.144	0.044

Random Components

Groups	Name	Variance	SD	ICC
**Horses**	(Intercept)	1.69	1.30	0.200
**Residual**		6.76	2.60	

Random Components

Groups	Name	Variance	SD	ICC

Note. Number of Obs: 32 , Number of groups: Horses 8

Post Hoc comparison: Time

Comparison							
Time	vs	Time	Difference	SE	t	df	p_{tukey}
T1	-	T2	-0.5750	1.30	-0.4422	21.0	0.970
T1	-	T3	-2.7625	1.30	-2.1247	21.0	0.178
T1	-	T4	-2.7875	1.30	-2.1439	21.0	0.172
T2	-	T3	-2.1875	1.30	-1.6825	21.0	0.357
T2	-	T4	-2.2125	1.30	-1.7017	21.0	0.348
T3	-	T4	-0.0250	1.30	-0.0192	21.0	1.000

FE WT13

Model Fit

Type	R^2	df	LRT X^2	p
Conditional	0.570	4	25.036	<.001
Marginal	0.507	3	25.036	<.001

Fixed Effects Omnibus Tests

	F	df	df (res)	p
**Time**	12.2	3	21.0	<.001

Parameter Estimates (Fixed coefficients)

				95% Confidence Intervals				
Names	Effect	Estimate	SE	Lower	Upper	df	t	p
(Intercept)	(Intercept)	6.972	0.351	6.250	7.694	7.00	19.839	<.001
Time1	T2 - T1	2.288	0.790	0.664	3.911	21.00	2.896	0.009

Parameter Estimates (Fixed coefficients)

Names	Effect	Estimate	SE	95% Confidence Intervals Lower	Upper	df	t	p
Time2	T3 - T1	-0.162	0.790	-1.786	1.461	21.00	-0.206	0.839
Time3	T4 - T1	-2.487	0.790	-4.111	-0.864	21.00	-3.150	0.005

Random Components

Groups	Name	Variance	SD	ICC
**Horses**	(Intercept)	0.364	0.604	0.127
**Residual**		2.495	1.580	

Note. Number of Obs: 32 , Number of groups: Horses 8

Post Hoc comparison: Time

Comparison							
Time	vs	Time	Difference	SE	t	df	p_{tukey}
T1	-	T2	-2.288	0.790	-2.896	21.0	0.040
T1	-	T3	0.162	0.790	0.206	21.0	0.997
T1	-	T4	2.487	0.790	3.150	21.0	0.023
T2	-	T3	2.450	0.790	3.102	21.0	0.026
T2	-	T4	4.775	0.790	6.046	21.0	<.001
T3	-	T4	2.325	0.790	2.944	21.0	0.036

FE T13T18

Model Fit

Type	R^2	df	LRT X^2	p
Conditional	0.470	4	21.897	<.001
Marginal	0.470	3	21.897	<.001

Fixed Effects Omnibus Tests

	F	df	df (res)	p
**Time**	9.17	3	28.0	<.001

Parameter Estimates (Fixed coefficients)

				95% Confidence Intervals				
**Names**	**Effect**	**Estimate**	**SE**	**Lower**	**Upper**	**df**	**t**	**p**
(Intercept)	(Intercept)	5.38	0.386	4.591	6.177	28.0	13.96	<.001
Time1	T2 - T1	3.21	1.091	0.970	5.455	28.0	2.94	0.006
Time2	T3 - T1	2.81	1.091	0.570	5.055	28.0	2.58	0.016
Time3	T4 - T1	-1.69	1.091	-3.930	0.555	28.0	-1.55	0.133

Random Components

Groups	Name	Variance	SD	ICC
**Horses**	(Intercept)	4.63e-15	6.80e-8	9.72e-16
**Residual**		4.76	2.18	

Note. Number of Obs: 32 , Number of groups: Horses 8

Post Hoc comparison: Time

Comparison							
**Time**	**vs**	**Time**	**Difference**	**SE**	**t**	**df**	p_{tukey}
T1	-	T2	-3.212	1.09	-2.944	28.0	0.031
T1	-	T3	-2.813	1.09	-2.578	28.0	0.070
T1	-	T4	1.688	1.09	1.547	28.0	0.424
T2	-	T3	0.400	1.09	0.367	28.0	0.983
T2	-	T4	4.900	1.09	4.491	28.0	<.001
T3	-	T4	4.500	1.09	4.124	28.0	0.002

FE T18L3

Model Fit

Type	R^2	df	LRT X^2	p
Conditional	0.503	4	20.484	<.001
Marginal	0.438	3	20.484	<.001

Fixed Effects Omnibus Tests

	F	df	df (res)	p
**Time**	9.11	3	21.0	<.001

Parameter Estimates (Fixed coefficients)

				95% Confidence Intervals				
**Names**	**Effect**	**Estimate**	**SE**	**Lower**	**Upper**	**df**	**t**	**p**
(Intercept)	(Intercept)	6.14	0.454	5.211	7.076	7.00	13.54	<.001
Time1	T2 - T1	3.66	1.040	1.525	5.800	21.00	3.52	0.002
Time2	T3 - T1	2.04	1.040	-0.100	4.175	21.00	1.96	0.063
Time3	T4 - T1	-1.38	1.040	-3.513	0.763	21.00	-1.32	0.200

Random Components

Groups	Name	Variance	SD	ICC
**Horses**	(Intercept)	0.565	0.751	0.115
**Residual**		4.325	2.080	

Note. Number of Obs: 32 , Number of groups: Horses 8

Post Hoc comparison: Time

Comparison							
**Time**	**vs**	**Time**	**Difference**	**SE**	**t**	**df**	p_{tukey}
T1	-	T2	-3.66	1.04	-3.52	21.0	0.010
T1	-	T3	-2.04	1.04	-1.96	21.0	0.235
T1	-	T4	1.38	1.04	1.32	21.0	0.560

Post Hoc comparison: Time

Comparison							
Time	vs	Time	Difference	SE	t	df	p_{tukey}
T2	-	T3	1.62	1.04	1.56	21.0	0.420
T2	-	T4	5.04	1.04	4.84	21.0	<.001
T3	-	T4	3.41	1.04	3.28	21.0	0.017

FE L3S

Model Fit

Type	R^2	df	LRT X^2	p
Conditional	0.140	4	4.865	0.301
Marginal	0.129	3	4.865	0.182

Fixed Effects Omnibus Tests

	F	df	df (res)	p
**Time**	1.55	3	21.0	0.231

Parameter Estimates (Fixed coefficients)

				95% Confidence Intervals				
Names	Effect	Estimate	SE	Lower	Upper	df	t	p
(Intercept)	(Intercept)	7.459	0.419	6.60	8.321	7.00	17.793	<.001
Time1	T2 - T1	-0.438	1.157	-2.82	1.941	21.00	-0.378	0.709
Time2	T3 - T1	0.887	1.157	-1.49	3.266	21.00	0.767	0.452
Time3	T4 - T1	-1.563	1.157	-3.94	0.816	21.00	-1.350	0.191

Random Components

Groups	Name	Variance	SD	ICC
**Horses**	(Intercept)	0.0673	0.259	0.0124
**Residual**		5.3552	2.314	

Random Components

Groups	Name	Variance	SD	ICC

Note. Number of Obs: 32 , Number of groups: Horses 8

Post Hoc comparison: Time

Comparison							
Time	vs	Time	Difference	SE	t	df	p_{tukey}
T1	-	T2	0.438	1.16	0.378	21.0	0.981
T1	-	T3	-0.887	1.16	-0.767	21.0	0.868
T1	-	T4	1.563	1.16	1.350	21.0	0.543
T2	-	T3	-1.325	1.16	-1.145	21.0	0.667
T2	-	T4	1.125	1.16	0.972	21.0	0.766
T3	-	T4	2.450	1.16	2.117	21.0	0.180

LB WT13

Model Fit

Type	R^2	df	LRT X^2	p
Conditional	0.302	4	8.668	0.070
Marginal	0.205	3	8.701	0.034

Fixed Effects Omnibus Tests

	F	df	df (res)	p
**Time**	3.04	3	21.0	0.052

Parameter Estimates (Fixed coefficients)

				95% Confidence Intervals				
Names	Effect	Estimate	SE	Lower	Upper	df	t	p
(Intercept)	(Intercept)	7.172	0.379	6.392	7.95	7.00	18.907	<.001
Time1	T2 - T1	1.400	0.861	-0.370	3.17	21.00	1.626	0.119
Time2	T3 - T1	1.350	0.861	-0.420	3.12	21.00	1.568	0.132

Parameter Estimates (Fixed coefficients)

Names	Effect	Estimate	SE	95% Confidence Intervals Lower	95% Confidence Intervals Upper	df	t	p
Time3	T4 - T1	-0.763	0.861	-2.532	1.01	21.00	-0.886	0.386

Random Components

Groups	Name	Variance	SD	ICC
**Horses**	(Intercept)	0.410	0.640	0.121
**Residual**		2.965	1.722	

Note. Number of Obs: 32 , Number of groups: Horses 8

Post Hoc comparison: Time

Comparison							
Time	vs	Time	Difference	SE	t	df	p_{tukey}
T1	-	T2	-1.4000	0.861	-1.6261	21.0	0.386
T1	-	T3	-1.3500	0.861	-1.5681	21.0	0.417
T1	-	T4	0.7625	0.861	0.8857	21.0	0.812
T2	-	T3	0.0500	0.861	0.0581	21.0	1.000
T2	-	T4	2.1625	0.861	2.5118	21.0	0.087
T3	-	T4	2.1125	0.861	2.4537	21.0	0.097

LB T13T18

Model Fit

Type	R^2	df	LRT X^2	p
Conditional	0.418	4	8.307	0.081
Marginal	0.096	3	5.280	0.152

Fixed Effects Omnibus Tests

	F	df	df (res)	p
**Time**	1.70	3	21.0	0.197

Parameter Estimates (Fixed coefficients)

				95% Confidence Intervals				
**Names**	**Effect**	**Estimate**	**SE**	**Lower**	**Upper**	**df**	**t**	**p**
(Intercept)	(Intercept)	3.253	0.289	2.659	3.847	7.00	11.258	<.001
Time1	T2 - T1	0.488	0.456	-0.450	1.425	21.00	1.069	0.297
Time2	T3 - T1	-0.512	0.456	-1.450	0.425	21.00	-1.124	0.274
Time3	T4 - T1	-0.212	0.456	-1.150	0.725	21.00	-0.466	0.646

Random Components

Groups	Name	Variance	SD	ICC
**Horses**	(Intercept)	0.460	0.678	0.356
**Residual**		0.832	0.912	

Note. Number of Obs: 32 , Number of groups: Horses 8

Post Hoc comparison: Time

Comparison							
**Time**	**vs**	**Time**	**Difference**	**SE**	**t**	**df**	**p_{tukey}**
T1	-	T2	-0.488	0.456	-1.069	21.0	0.712
T1	-	T3	0.512	0.456	1.124	21.0	0.679
T1	-	T4	0.212	0.456	0.466	21.0	0.966
T2	-	T3	1.000	0.456	2.193	21.0	0.158
T2	-	T4	0.700	0.456	1.535	21.0	0.435
T3	-	T4	-0.300	0.456	-0.658	21.0	0.912

LB T18L3

Model Fit

Type	R^2	df	LRT X^2	p
Conditional	0.270	4	6.409	0.171
Marginal	0.146	3	6.251	0.100

Fixed Effects Omnibus Tests

	F	df	df (res)	p
**Time**	2.06	3	21.0	0.136

Parameter Estimates (Fixed coefficients)

				95% Confidence Intervals				
Names	Effect	Estimate	SE	Lower	Upper	df	t	p
(Intercept)	(Intercept)	3.416	0.219	2.966	3.866	7.00	15.603	<.001
Time1	T2 - T1	0.725	0.478	-0.258	1.708	21.00	1.517	0.144
Time2	T3 - T1	0.762	0.478	-0.220	1.745	21.00	1.595	0.126
Time3	T4 - T1	-0.175	0.478	-1.158	0.808	21.00	-0.366	0.718

Random Components

Groups	Name	Variance	SD	ICC
**Horses**	(Intercept)	0.155	0.394	0.145
**Residual**		0.914	0.956	

Note. Number of Obs: 32 , Number of groups: Horses 8

Post Hoc comparison: Time

Comparison							
Time	vs	Time	Difference	SE	t	df	p_{tukey}
T1	-	T2	-0.7250	0.478	-1.5168	21.0	0.446
T1	-	T3	-0.7625	0.478	-1.5952	21.0	0.403
T1	-	T4	0.1750	0.478	0.3661	21.0	0.983

Post Hoc comparison: Time

Comparison							
Time	vs	Time	Difference	SE	t	df	p_{tukey}
T2	-	T3	-0.0375	0.478	-0.0785	21.0	1.000
T2	-	T4	0.9000	0.478	1.8829	21.0	0.265
T3	-	T4	0.9375	0.478	1.9614	21.0	0.234

LB L3S

Model Fit

Type	R^2	df	LRT X^2	p
Conditional	0.246	4	6.297	0.178
Marginal	0.151	3	6.283	0.099

Fixed Effects Omnibus Tests

	F	df	df (res)	p
**Time**	2.08	3	21.0	0.134

Parameter Estimates (Fixed coefficients)

				95% Confidence Intervals				
Names	Effect	Estimate	SE	Lower	Upper	df	t	p
(Intercept)	(Intercept)	2.728	0.163	2.3928	3.063	7.00	16.722	<.001
Time1	T2 - T1	0.425	0.377	-0.3490	1.199	21.00	1.129	0.272
Time2	T3 - T1	0.862	0.377	0.0885	1.637	21.00	2.290	0.032
Time3	T4 - T1	0.125	0.377	-0.6490	0.899	21.00	0.332	0.743

Random Components

Groups	Name	Variance	SD	ICC
**Horses**	(Intercept)	0.0711	0.267	0.111
**Residual**		0.5672	0.753	

Random Components

Groups	Name	Variance	SD	ICC

Note. Number of Obs: 32 , Number of groups: Horses 8

Post Hoc comparison: Time

Comparison							
Time	vs	Time	Difference	SE	t	df	p_{tukey}
T1	-	T2	-0.425	0.377	-1.129	21.0	0.676
T1	-	T3	-0.862	0.377	-2.290	21.0	0.132
T1	-	T4	-0.125	0.377	-0.332	21.0	0.987
T2	-	T3	-0.438	0.377	-1.162	21.0	0.657
T2	-	T4	0.300	0.377	0.797	21.0	0.855
T3	-	T4	0.738	0.377	1.958	21.0	0.235
